# Blockade of ZMIZ1‐GATA4 Axis Regulation Restores Youthfulness to Aged Cartilage

**DOI:** 10.1002/advs.202404311

**Published:** 2025-03-05

**Authors:** Jiho Nam, Hyunmin Woo, Jihye Yang, Seok Jung Kim, Kwang Pyo Lee, Ji Hoon Yu, Tae Joo Park, Seong‐il Eyun, Siyoung Yang

**Affiliations:** ^1^ Department of Biological Science Sungkyunkwan University Suwon 16419 Republic of Korea; ^2^ Department of Life Science Chung‐Ang University Seoul 06974 Republic of Korea; ^3^ Department of Orthopedic Surgery Uijeongbu St. Mary's Hospital The Catholic University of Korea College of Medicine Uijeongbu 11765 Republic of Korea; ^4^ Aging Research Center Korea Research Institute of Bioscience and Biotechnology (KRIBB) Daejeon 34141 Republic of Korea; ^5^ New Drug Development Center Daegu‐Gyeongbuk Medical Innovation Foundation (K‐MEDI hub) Daegu 41061 Republic of Korea; ^6^ Department of Biological Sciences Ulsan National Institute of Science and Technology (UNIST) Ulsan 44919 Republic of Korea

**Keywords:** cartilage restoration, osteoarthritis, senescence, ZMIZ1‐GATA4 axis

## Abstract

Susceptibility to cartilage degeneration increases in an age‐dependent manner and older cartilage exhibits increased catabolic factor expression leading to osteoarthritis (OA). While inhibition of cellular senescence can prevent age‐related diseases, the understanding of the regulators governing cartilage senescence and the potential for senolytic intervention remains limited. Here, in vitro and in vivo results are reported, demonstrating for the first time that the transcriptional regulator, ZMIZ1, is upregulated in aged and OA cartilage, and that it acts through GATA4 to accelerate chondrocyte senescence and trigger cartilage deterioration. Furthermore, it is shown that K‐7174 interferes with the ZMIZ1‐GATA4 interaction and effectively hampers cartilage senescence and OA. It is proposed that inhibition of the ZMIZ1‐GATA4 axis could be a valuable strategy for eliminating senescent chondrocytes and impeding OA development and that the relevant inhibitor, K‐7174, could potentially be developed as a senolytic drug for managing cartilage senescence and age‐related degeneration.

## Graphical abstract

The blockade of ZMIZ1‐GATA axis can be an effective therapeutic approach for treating OA. Functional inhibition of the ZMIZ1‐GATA axis through K‐7174 has the effect of suppressing cartilage damage and cellular senescence at the chondrocyte and cartilage tissue levels. The strategy of blocking age‐related OA using K‐7174 may be a potential treatment method.

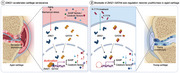

## 1 Introduction

The socioeconomic cost of osteoarthritis (OA) is large and on the rise.^[^ ^1^, ^2^, ^3^, ^4^ ^]^ Chondrocyte senescence accelerates cartilage degeneration and OA,^[^ ^5^, ^6^, ^7^, ^8^ ^]^ whereas the clearance of senescent chondrocytes can protect against cartilage degeneration and OA development.^[^ ^9^, ^10^, ^11^ ^]^ Senescent cells can be identified by their expression of *Glb1* encoded senescence‐associated β‐galactosidase (SA‐β‐gal), and p16.^[^ ^8^, ^12^, ^13^, ^14^ ^]^ These cells undergo morphological changes and exhibit the senescence‐associated secretory phenotype (SASP), which is characterized by the secretion of various inflammatory cytokines and matrix metalloproteinases (MMPs). As cartilage ages, the buildup of senescent chondrocytes increases SASP‐mediated cartilage/extracellular matrix (ECM) degradation and inflammation through the up‐regulation of MMPs and cyclooxygenase 2 (COX2), respectively.^[^ ^15^, ^16^ ^]^ The accumulation of MMPs induced by pro‐inflammatory cytokines is another positive marker for senescence in OA chondrocytes.^[^ ^17^ ^]^ Most phenotypic manifestations of chondrocyte senescence appear to be induced in OA via the transcriptional regulator‐based modulation of transcription initiation.^[^ ^18^ ^]^ The dysregulation of transcriptional regulators is also associated with cellular senescence and the induction of age‐related diseases.^[^ ^19^ ^]^ We can logically link these symptoms to the age‐dependent etiology of OA. Although transcriptional regulators are considered to be attractive targets for senolytic drugs (agents that selectively clear senescent cells), little is known about the transcriptional regulators involved in cartilage senescence and age‐dependent catabolic factor expression.

ZMIZ1 (zinc finger, MIZ‐type containing 1) is a transcriptional regulator that is involved in various cellular processes, including gene transcription, DNA repair, and cell cycle regulation.^[^ ^20^, ^21^, ^22^ ^]^ It contains several domains, including a C2H2 zinc finger domain, a SP‐RING (SplA and ryanodine receptor) domain, and a MIZ (Msx‐interacting zinc finger) domain.^[^ ^23^, ^24^ ^]^

Although it is reportedly associated with the development of cancer and various degenerative diseases,^[^ ^20^, ^21^, ^22^, ^23^ ^]^ no previous study has directly demonstrated its role in cell senescence. Studies have shown that there is a close relationship between cell senescence and the development of degenerative diseases and cancer^[^ ^25^, ^26^ ^]^ and that chondrocyte senescence directly contributes to OA pathogenesis.^[^ ^27^ ^]^ It seems logical that ZMIZ1 could be associated with both OA pathogenesis and chondrocyte senescence. However, its roles in chondrocyte senescence and cartilage degeneration are largely unknown.

Here, we report experiments performed in vitro and in cartilage‐specific *Zmiz1* transgenic mice in vivo. Our results show for the first time that ZMIZ1 is involved in chondrocyte senescence and cartilage degeneration. Blockade of ZMIZ1 expression by intra‐articular (IA) injection of Ad‐sh*Zmiz1* protects against cartilage degeneration through inhibition of chondrocyte senescence. Progression of chondrocyte senescence and osteoarthritic cartilage destruction requires the interaction of ZMIZ1 with the transcription factor GATA4, whereas K‐7174, which inhibits the ZMIZ1‐GATA interaction, promotes the removal of senescent chondrocytes and inhibits OA pathogenesis. Based on our findings, we propose that blockade of the ZMIZ1‐GATA4 axis may be an attractive senolytic strategy against cartilage degeneration and OA development.

## 2 Results

### 2.1 ZMIZ1 Accelerates Cartilage Senescence and Cartilage Destruction

Although it is challenging to monitor spontaneous osteoarthritic cartilage destruction in aged C57BL6 mice, aged mice are more sensitive than young mice to surgery‐induced OA.^[^ ^28^ ^]^ To determine whether aged cartilage exhibits increased susceptibility to OA phenotypes, we compared cartilage tissues from 12‐, 22‐, and 60‐week‐old mice. Histological staining showed increased cartilage surface wear and thickening of the subchondral bone plate in 60‐week‐old mice compared to their younger counterparts (Figure S1A,B, Supporting Information). Micro‐CT results showed that as the mouse age increased, the subchondral bone became deformed and thickened and the proportion of the thick part increased (Figure S1C, Supporting Information). Additionally, senescence marker such as GLB1, p16, and p21 expression levels increased with age in 12‐, 22‐, and 60‐week‐old mice. (Figure S2, Supporting Information). To provide information on mechanical and compositional cartilage traits, we analyzed the elastic modulus of the cartilage using the bioindentation technique. Sixty‐week‐old mouse cartilage showed a lower elastic modulus than 12‐week‐old mouse cartilage (Figure S1D, Supporting Information). These results suggest that the aged cartilage itself is worn down to some extent, and thus may be more susceptible to severe OA. DMM (destabilization of the medial meniscus) surgery in mice provides a reproducible and slow‐progressing disease model that resembles the development of human OA.^[^ ^29^ ^]^ To determine whether OA stimulation causes more severe cartilage damage in 60‐week‐old mice, we performed DMM surgery on mice at 12 and 60 weeks of age (Figure S3, Supporting Information). In mice given DMM surgery at 12 weeks old, cartilage damage gradually appeared at ≈7 weeks post‐surgery; in those given DMM surgery at 60 weeks old, in contrast, damage was first apparent at 4 weeks post‐surgery. Similarly, cartilage damage and subchondral bone thickening were induced at an earlier point post‐surgery in old mice (Figure S1E,F, Supporting Information). These data suggested that susceptibility to OA progression is sensitively related to cartilage age.

Cellular senescence is accompanied by a distinct secretory phenotype called the senescence‐associated secretory phenotype (SASP), which involves secretion of factors such as p16 and senescence‐associated β‐galactosidase (SA‐β‐gal). But we know little about the specific transcriptional regulator(s) involved in the senescence of chondrocytes and cartilage.

**Figure 1:**
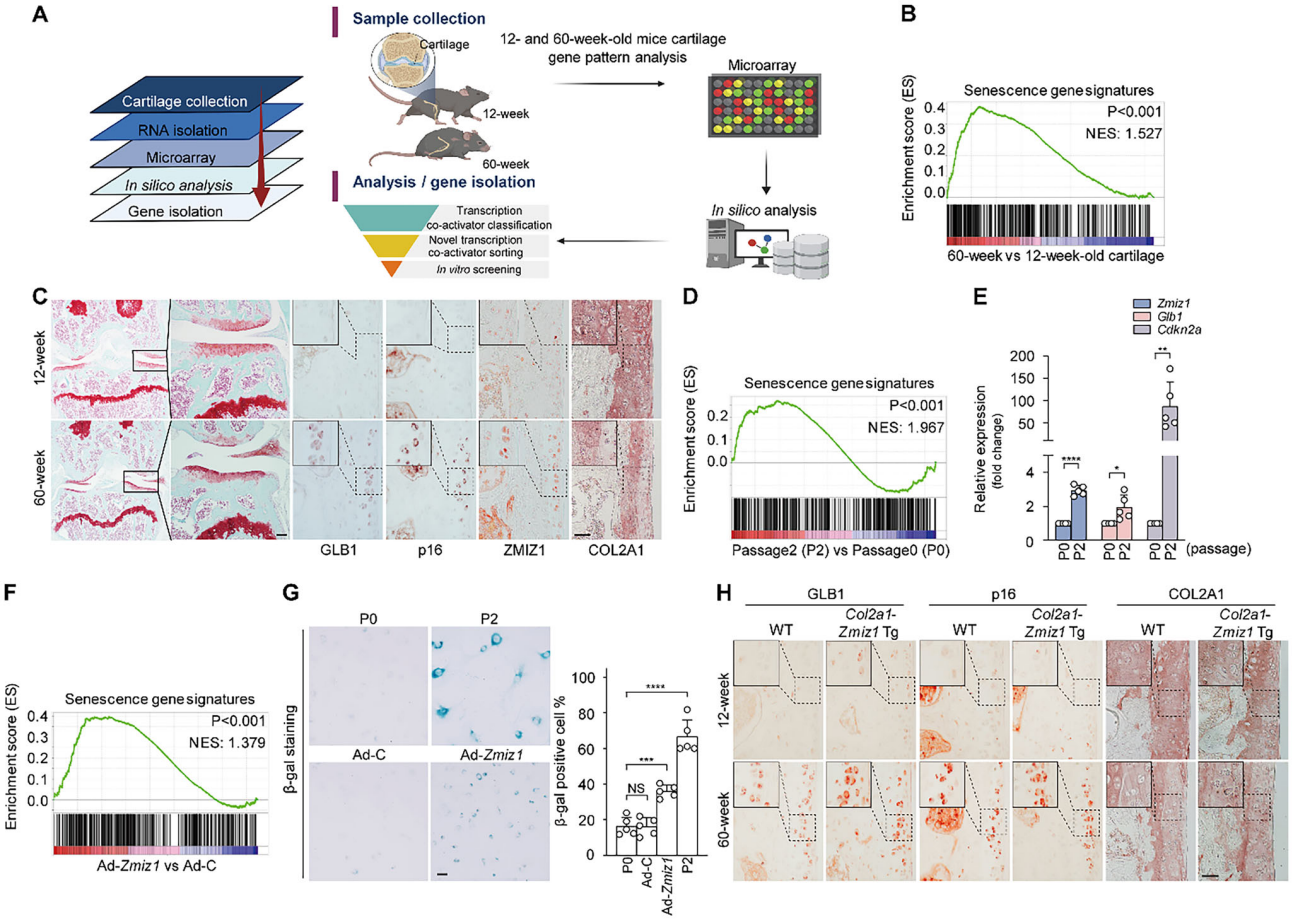
Identification of ZMIZ1 as a senescence‐related transcriptional regulator. (A) Schematic showing the strategy used to identify ZMIZ1 as a candidate cartilage senescence‐ and OA‐related transcriptional regulator. The left panel shows a simplified overall schematic diagram. The right panel shows a detailed schematic diagram of the gene‐sorting strategy. (B) Correlation analysis of senescence‐related genes in chondrocytes from 60‐ and 12‐week‐old mice, as assessed using GSEA. (C) Histological analysis of the cartilage and protein levels of GLB1, p16, ZMIZ1, and COL2A1 in 12‐ and 60‐week‐old mouse cartilage (*n* = 5; Safranin O staining image scale bar: 100 µm, immunohistochemistry scale bar: 50 µm). (D) GSEA analysis of senescence gene signatures in gene sets from passage 2 and passage 0 chondrocytes. (E) The expression levels of *Zmiz1*, *Glb1*, and *Cdkn2a* in passaged chondrocytes, as assessed by qRT‐PCR (*n* = 5; two‐tailed *t*‐test). (F) GSEA‐based correlation analysis between *Zmiz1* overexpression and senescence. (G) β‐gal staining in passaged (upper panel) and Ad‐*Zmiz1* (800 MOI)‐infected (lower panel) mouse chondrocytes and quantified percentage of stained cells (*n* = 5; scale bar: 100 µm; one‐way ANOVA with Dunnett's multiple comparison test). (H) Detection of GLB1, p16, and COL2A1 in cartilage of 12‐ and 60‐week‐old WT and *Col2a1*‐*Zmiz1* Tg mice (*n* = 5; scale bar: 50 µm). The presented magnifications are as follows: Safranin O staining, 100 × IHC, 400 × and β‐gal staining, 100 ×. The area enlarged to provide a magnified local view, emphasizing the staining for IHC. Values are expressed as the mean ± SEM. *p < 0.05, **p < 0.01, ***p < 0.001, ****p < 0.0001. NS is non‐significant.

To address the lack of knowledge regarding the transcriptional regulators responsible for regulating SASP factors in the senescence of chondrocyte and cartilage, we used microarray analysis to compare 12‐ and 60‐week‐old mouse cartilage. To identify transcriptional regulators specific to 60‐week‐old cartilage, we subjected our microarray results to IPA (Ingenuity Pathway Analysis) and sorted genes as shown in the schematic diagram (**Figure** **1**A). We hypothesized that a novel OA pathogenic transactional regulator would need to be expressed under both cartilage degeneration and chondrocyte senescence. We thus screened for regulators in vitro using OA‐ and chondrocyte senescence‐mimicking conditions, namely IL‐1β‐treated chondrocytes and subcultured chondrocytes, respectively. We selected *Zmiz1*, which was expressed only under both conditions, as a strong candidate for a transcriptional regulator involved in the induction of OA and senescence (Figure 1A and Table S1, Supporting Information). GSEA (Gene Set Enrichment Analysis) demonstrated that the gene set up‐regulated in 60‐week‐old cartilage was enriched for genes related to senescence (Figure 1B). We previously confirmed the elevated protein expression of several functionally relevant genes, such as those encoding SASP factors (Figure S2, Supporting Information). These results indicate that our array data obtained from 60‐week‐old cartilage should be a reliable platform for studying chondrocyte senescence. Consistent with our hypothesis regarding the involvement of ZMIZ1 in cartilage senescence, the protein level of ZMIZ1 was increased in senescent 60‐week‐old cartilage tissue, whereas that of the anabolic factor, COL2A1, was decreased (Figure 1C; Figure S4A, Supporting Information). Although a previous report indicated that a high number of chondrocyte passages can be used to generate an in vitro senescence‐mimicking condition,^[^ ^6^, ^30^ ^]^ we found that passage 2 (P2) chondrocytes exhibited sufficient signs of senescence, including up‐regulation of aging factors and down‐regulation of anabolic factors (Figure S5, Supporting Information). Through GSEA analysis, we confirmed that chondrocytes subcultured to P2 exhibited profiles consistent with senescence (Figure 1D). Under these in vitro senescence‐mimicking conditions, we observed significant up‐regulation of *Zmiz1* and SASP factors (Figure 1E,G; upper panel, Figure S5, Supporting Information). To further assess whether *Zmiz1* regulates SASP factor expression for chondrocyte senescence, we used an adenovirus delivery system.^[^ ^31^, ^32^, ^33^ ^]^ GSEA showed that Ad‐*Zmiz1* infection of chondrocytes was correlated with senescence (Figure 1F), and β‐gal staining showed that Ad‐*Zmiz1* infection increased the number of senescent chondrocytes (Figure 1G; lower panel). Given that chondrocyte dedifferentiation, which is marked by the loss of anabolic factor expression, leads to chondrocyte senescence,^[^ ^17^ ^]^ we also assessed the mRNA expression levels of the major anabolic factors, *Col2a1*, *Acan*, and *Sox9*, and found that they were decreased by Ad‐*Zmiz1* infection (Figure S6, Supporting Information). These data strongly support the idea that ZMIZ1 overexpression induces chondrocyte senescence.

We next generated cartilage‐specific ZMIZ1 transgenic (*Col2a1*‐*Zmiz1* Tg) mice^[^ ^29^ ^]^ with cartilage‐specific overexpression of ZMIZ1 to assess the in vivo function of *Zmiz1* in cartilage senescence (Figure S7A, Supporting Information). We did not observe any size difference between WT and *Col2a1*‐*Zmiz1* Tg mice (Figure S7B, Supporting Information). However, similar to 60‐week‐old WT mice, *Col2a1*‐*Zmiz1* Tg mice exhibited up‐regulation of representative SASP factors (GLB1 and p16) and down‐regulation of the anabolic factor, COL2A1 (Figure 1H; Figure S4B, Supporting Information).

### 2.2 Overexpression of Zmiz1 Leads to Upregulation of OA Gene Signatures

**Figure 2:**
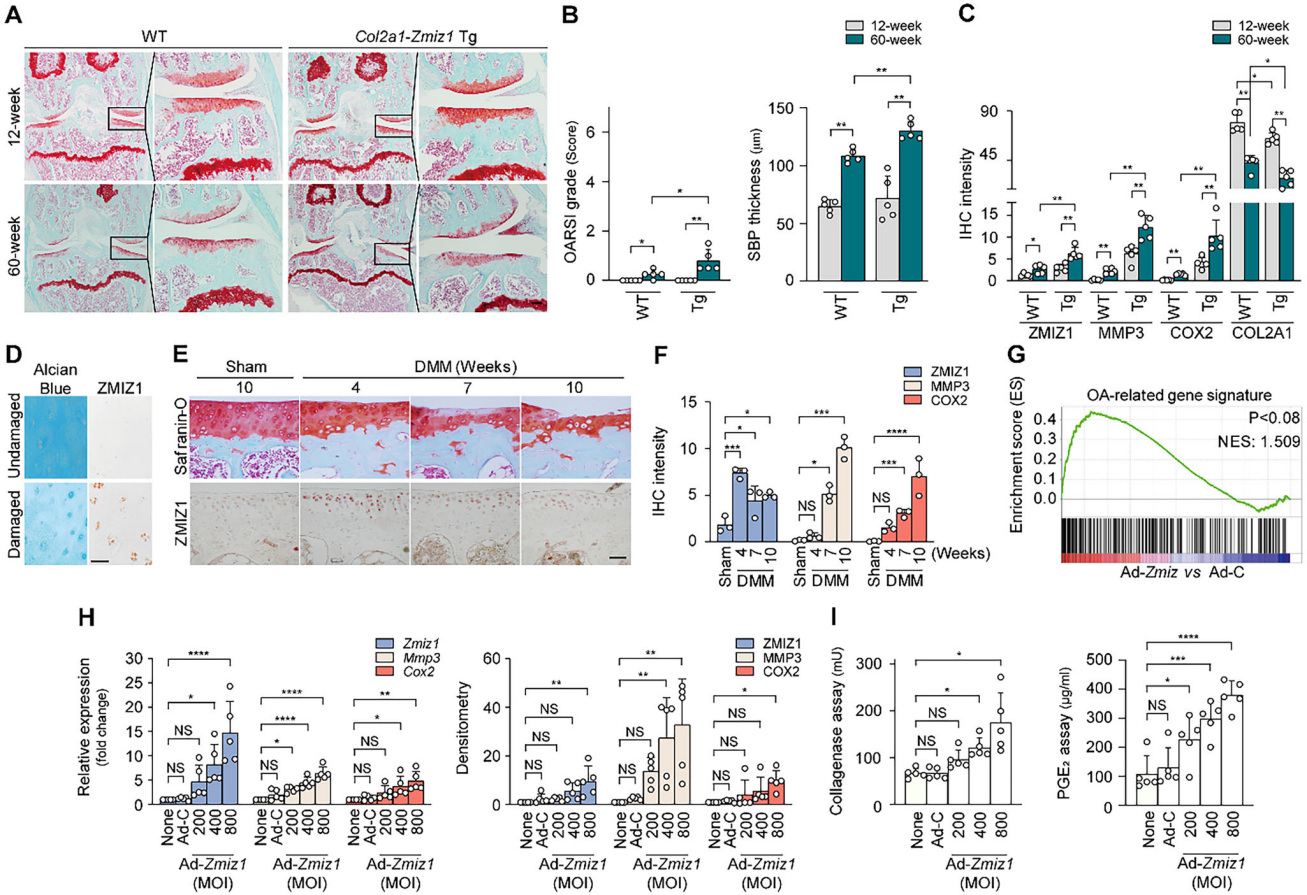
ZMIZ1 overexpression in cartilage regulates OA. (A,B) Cartilage tissues of *Col2a1*‐*Zmiz1* Tg and WT mice were compared. (A) Cartilage tissues of 12‐week‐old young mice and 60‐week‐old aged mice of each genotype were analyzed with Safranin‐O staining (*n* = 5; scale bar: 100 µm). (B) The degree of tissue damage was measured through OARSI scoring and SBP thickness determination (*n* = 5; Mann–Whitney U test). (C) Quantification of ZMIZ1, MMP3, and COX2 expression levels in cartilage tissues from mice of the indicated genotype and age (*n* = 5; Mann–Whitney U test). (D) ZMIZ1 expression in damaged human cartilage tissues (scale bar: 50 µm). (E and F) Expression of ZMIZ1 in mouse cartilage at the indicated weeks after DMM surgery. (E) Histological images of mouse cartilage (*n* = 3; scale bar: 50 µm) and (F) quantification of ZMIZ1, MMP3, and COX2 expression levels (*n* = 3; one‐way ANOVA with Dunnett's multiple comparison test). (G) GSEA analysis of OA‐related gene expression patterns in chondrocytes overexpressing *Zmiz1* due to infection with Ad‐*Zmiz1* (800 MOI). (H) Quantification of each factor by qRT‐PCR (left) and WB (Western blot) densitometry (right) of cells infected with the indicated multiplicity of infection (MOI) of Ad‐*Zmiz1* (*n* = 5; one‐way ANOVA with Dunnett's multiple comparison test). (I) Collagenase activity (left) and PGE_2_ production (right) were quantified in chondrocytes infected with Ad‐*Zmiz1* (*n* = 5; one‐way ANOVA with Dunnett's multiple comparison test). The presented magnifications are as follows: Safranin O, 100 × and IHC, 400 ×. Values are expressed as the mean ± SEM. *p < 0.05, **p < 0.01, ***p < 0.001 and ****p < 0.0001. NS is non‐significant.

Although mouse cartilage does not deteriorate per se with aging, histological staining reportedly showed that aged cartilage exhibits slight decreases in glycosaminoglycan and cartilage layers, and an uneven surface.^[^ ^28^ ^]^ In the present work, Safranin‐O staining and OARSI grading showed that the cartilage of 60‐week‐old *Col2a1*‐*Zmiz1* Tg mice exhibited increased age‐related phenotypes compared to that of WT mice (**Figure** **2**A,B). Examination of catabolic factor expression in 12‐ and 60‐week‐old *Col2a1*‐*Zmiz1* Tg and WT mice revealed that older mice of both genotypes showed increased catabolic factor expression compared to the corresponding younger mice (Figure 2C; Figure S8A, Supporting Information). These in vivo results show that ZMIZ1 overexpression worsens OA in aged mice. To explore whether there was a clinical correlation between ZMIZ1 and OA, we assessed ZMIZ1 expression levels in human and mouse OA cartilage. We found that ZMIZ1 was highly expressed in human OA cartilage compared to intact cartilage (Figure 2D; Figure S8B, Supporting Information). In WT mice given DMM surgery, COL2A1 expression gradually decreased post‐surgery; MMP3 and COX2 gradually increased with significance first reached at 7 weeks post‐surgery; and ZMIZ1 was highly expressed at 4 weeks post‐surgery with lower but still significantly increased levels seen at 7‐ and 10‐weeks post‐surgery related to the sham‐operated control (Figure 2E,F; Figure S8C,D, Supporting Information). Likewise, in chondrocytes stimulated to undergo OA pathogenesis by IL‐1β, TNF‐α, or IL‐17, the up‐regulation of *Zmiz1* preceded that of catabolic factors (Figure S8E, Supporting Information). These data strongly support the notion that ZMIZ1 may regulate catabolic factor expression in chondrocytes stimulated to enter OA pathogenesis.

We next assessed whether ZMIZ1 might play a role in osteoarthritic cartilage destruction. GSEA revealed that the gene set up‐regulated by Ad‐*Zmiz1* infection was positively related to a previously reported OA gene signature (Figure 2G). The OA‐related genes up‐regulated by Ad‐*Zmiz1* infection included those encoding MMP3 and COX2, which function as main factors of cartilage destruction and inflammation, respectively.^[^ ^31^, ^34^ ^]^ Our results showed that Ad‐*Zmiz1* infection increased the expression levels of the OA catabolic factors, MMP3 and COX2, at the transcript and protein levels in chondrocytes, and that ZMIZ1 overexpression did not trigger apparent cytotoxicity (Figure 2H; Figure S8F,G, Supporting Information). OA cartilage was reported to exhibit increased collagenase activity and PGE_2_ production, which could lead to the degradation of various ECM components through the expression of MMP3 and COX2.^[^ ^35^ ^]^ Here, we found that Ad‐*Zmiz1* infection at the indicated MOI led to increases in collagenase activity and PGE_2_ production (Figure 2I). These results suggest that ZMIZ1 may work as a master transcriptional regulator leading to OA pathogenesis.

### 2.3 Osteoarthritic Cartilage Destruction is Suppressed by ZMIZ1 Knockdown

**Figure 3:**
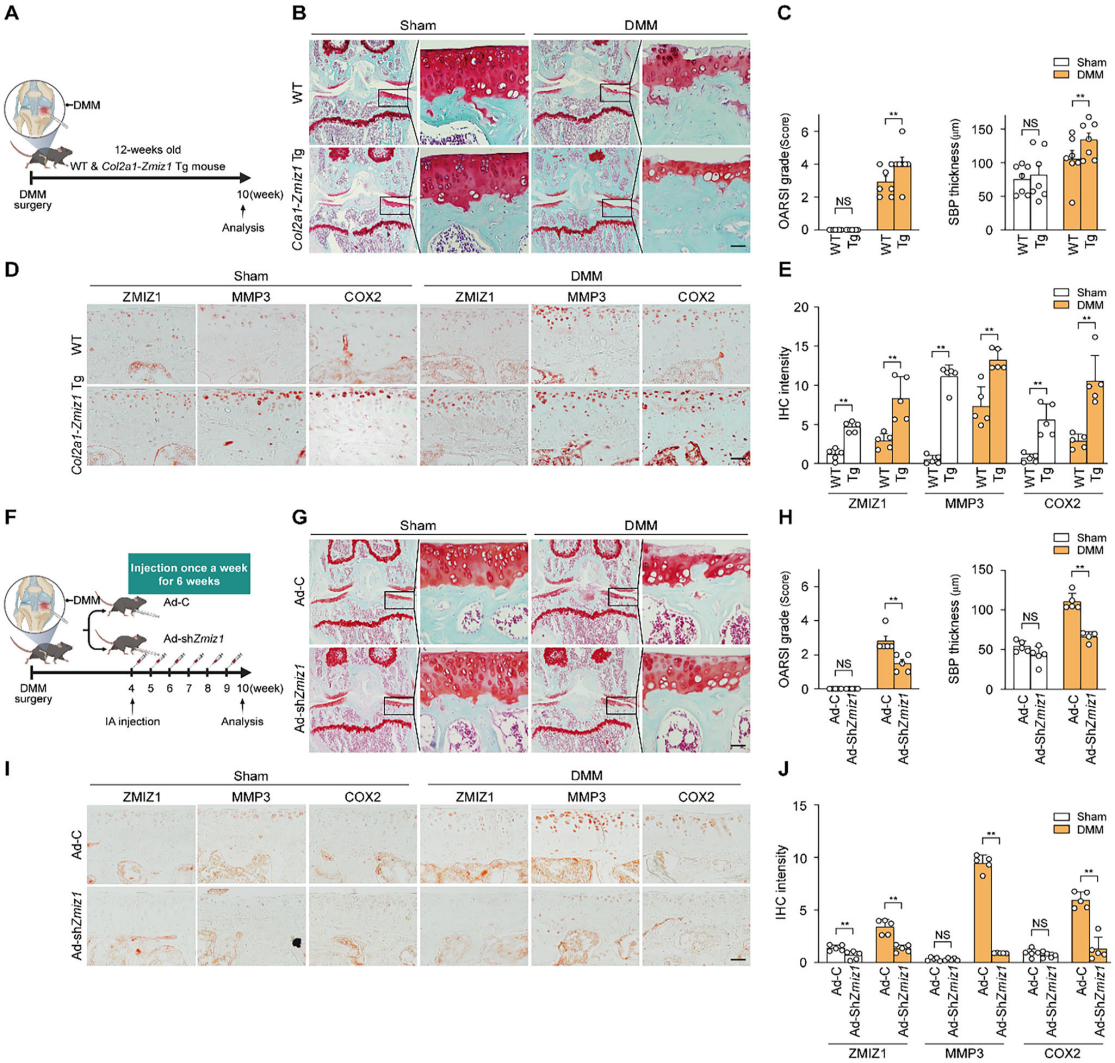
ZMIZ1 overexpression induces OA pathogenesis. The degree of cartilage tissue damage induced by DMM surgery was assessed relative to the expression of ZMIZ1. (A) Schematic diagram of the DMM surgery protocol. (B) Cartilage destruction induced by DMM surgery was assessed using Safranin‐O staining in *Col2a1*‐*Zmiz1* Tg and WT mice (*n* = 7; scale bar: 50 µm). (C) Cartilage damage was assessed by OARSI grade and SBP thickness (*n* = 7; Mann–Whitney U test). (D,E) ZMIZ1, MMP3, and COX2 immunostaining and quantification in DMM‐induced *Col2a1*‐*Zmiz1* Tg and WT mice (*n* = 5; scale bar: 50 µm; Mann–Whitney U test). (F) Schematic diagram of knee joint injection schedule in DMM‐operated mice. (G,H) Ad‐sh*Zmiz1* injected cartilage was stained with Safranin‐O and OA manifestations were assessed by scoring of OARSI grade and SBP thickness (*n* = 5; scale bar: 50 µm; Mann–Whitney U test). (I,J) Immunohistochemical analysis (left) and quantification (right) of ZMIZ1, MMP3, and COX2 in virus‐injected cartilage (*n* = 5; scale bar: 50 µm; Mann–Whitney U test). Safranin O images were taken at magnifications of 100 × and 400 ×. IHC images were taken at 400 ×. Values are expressed as the mean ± SEM. **p < 0.01. NS is non‐significant.

Mechanical stress and other inducers of OA reportedly trigger sensitive regulation of age‐dependent genes and alter the expression levels of catabolic factors, leading to OA pathogenesis.^[^ ^36^, ^37^ ^]^ Here, we assessed whether an OA‐mimicking condition would show enhanced OA pathogenesis in our *Zmiz1*‐ overexpressing in vitro and in vivo models. We first performed Ad‐*Zmiz1* infection with or without IL‐1β treatment in chondrocytes. *Mmp3* and *Cox2* expression were increased in IL‐1β‐treated chondrocytes with Ad‐*Zmiz1* infection, compared to the corresponding non‐infected chondrocytes (Figure S9A, Supporting Information). When OA was induced by DMM surgery as shown in the schematic diagram (**Figure** **3**A), *Col2a1*‐*Zmiz1* Tg mice showed more severe cartilage destruction and OA manifestations than WT mice (Figure 3B,C). Immunohistochemical analysis indicated that the expression levels of ZMIZ1, MMP3, and COX2 were increased in DMM‐induced *Col2a1*‐*Zmiz1* Tg mice compared to their WT counterparts, whereas the level of COL2A1 was decreased (Figure 3D,E; Figure S10A, Supporting Information). Thus, as seen in aged mice, OA catabolic factor expression was higher in the Tg mouse model itself, and these mice exhibited a greater degree of cartilage damage following DMM surgery compared to WT mice.

We also assessed the effects of *Zmiz1* knockdown in vitro and in vivo. The transcript and protein levels of catabolic factors in IL‐1β treated chondrocytes were decreased by *Zmiz1* knockdown (Figure S9B,C, Supporting Information). Similarly, when DMM‐induced OA model mice were injected Ad‐*Zmiz1* shRNA, which suppresses *Zmiz1* expression (see schematic diagram, Figure 3F), the mice exhibited reduction of subsequent cartilage damage (Figure 3G,H) and catabolic factor expression, along with recovery of COL2A1 expression (Figure 3I,J; Figure S10B, Supporting Information). Furthermore, similar to our findings in Ad‐*Zmiz1* shRNA‐infected young mice, *Zmiz1* knockdown in DMM‐induced old mice decreased cartilage destruction and down‐regulated several catabolic factors while up‐regulating anabolic factors (Figure S11, Supporting Information). These loss‐of‐function results suggest that ZMIZ1 plays an important role in the induction of OA.

To assess the expression levels of SASP factors in an in vivo model, we used *Col2a1*‐*Zmiz1* Tg and Ad‐*Zmiz1* shRNA‐infected mice with or without DMM surgery. Consistent with the above‐described observations regarding catabolic factor expression in mouse OA models in vivo (Figure 3D,E), SASP factors were further increased in *Col2a1*‐*Zmiz1* Tg mice (Figure S12A, Supporting Information), but this was reduced by Ad‐*Zmiz1* shRNA infection‐mediated knockdown of *Zmiz1* in both young and old mice (Figure S12B,C, Supporting Information). These observations suggest that ZMIZ1 may function as a transcriptional regulator of both chondrocyte senescence and OA progression.

### 2.4 ZMIZ1 Acts Together with GATA4 to Regulate Cartilage Damage and Senescence

**Figure 4:**
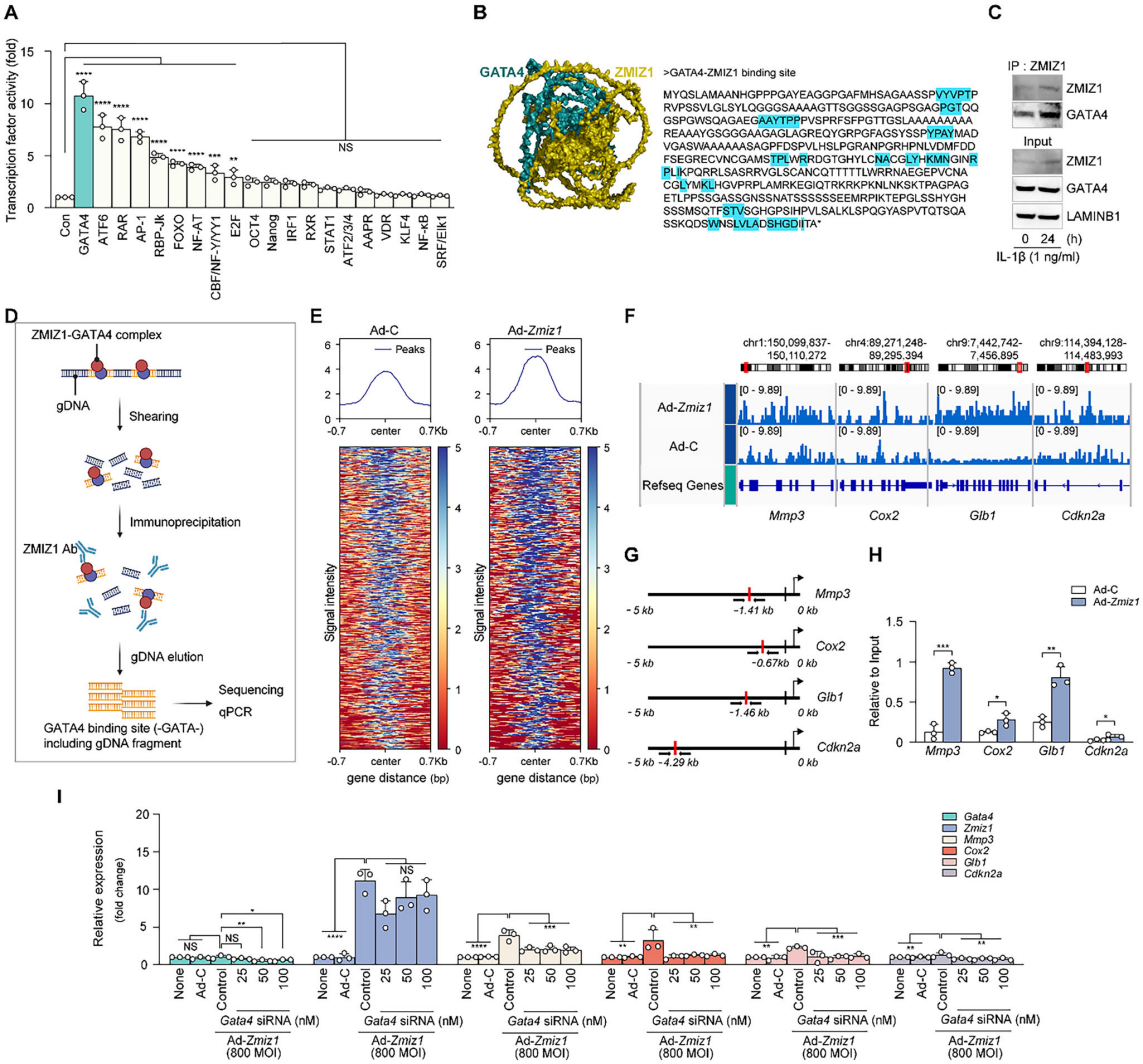
Chondrocyte senescence and OA‐related damage are regulated by the ZMIZ1‐GATA4 axis. (A) Transcription factor array analyses were performed using a Cignal 45‐Pathway Reporter Array (*n* = 3; one‐way ANOVA with Dunnett's multiple comparison test). (B) Computational docking models for ZMIZ1 (olive) and the transcription factor, GATA4 (cyan), were predicted with Alpha Fold2 and ClusPro 2.0. The predicted ZMIZ1‐binding sites on the GATA4 amino acid sequence (a.a.: 1–442) are shown. (C) Co‐IP results to confirm ZMIZ1 and GATA4 interaction (*n* = 5). (D) Schematic diagram showing the workflow for our ChIP experiment. (E) Heat maps for the signal intensities of GATA4‐binding sites in fragments collected from ChIP of Ad‐C and Ad‐*Zmiz1* groups (800 MOI). (F) Peak enrichments for the indicated genes in the Ad‐C and Ad‐*Zmiz1* groups. (G,H) Schematic showing the GATA4‐binding site at each indicated promoter region (G) and quantification of the expression level of each gene in the indicated group (H) (*n* = 3, by Mann–Whitney U test). (I) qRT‐PCR‐based quantification of the indicated Ad‐*Zmiz1* (800 MOI)‐regulated genes in *Gata4‐*knockdown chondrocytes (*n* = 3; one‐way ANOVA with Dunnett's multiple comparison test). Values are expressed as the mean ± SEM. *p < 0.05, **p < 0.01, ***p < 0.001 and ****p < 0.0001. NS is non‐significant.

ZMIZ1 contains a transcriptional activation domain and can bind to other transcription factors.^[^ ^22^ ^]^ In an effort to identify one or more binding partners of ZMIZ1, we performed a transcription factor array analysis. We found that the transcription factor, GATA4, was highly regulated by Ad‐*Zmiz1* infection in chondrocytes (**Figure** **4**A). Protein structural homology modeling predicted that ZMIZ1 can directly bind to GATA4 (Figure 4B). Furthermore, when IL‐1β treatment was used to trigger overexpression of endogenous ZMIZ1 in chondrocytes, co‐IP analysis showed that the endogenous ZMIZ1 directly interacted with the endogenous GATA4 (Figure 4C). These results demonstrate that ZMIZ1 directly binds to GATA4.

To assess the connection of GATA4 to OA and chondrocyte senescence, we first examined GATA4 protein expression levels. We found that the protein level of GATA4 was increased in both human and mouse OA cartilage related to non‐OA control samples, as well as in *Col2a1‐Zmiz1* Tg old mice compared to corresponding WT control mice (Figure S13A, Supporting Information). As previous reports demonstrated that H_2_O_2_ induces chondrocyte senescence,^[^ ^38^ ^]^ we further examined the expression levels of *Zmiz*1, *Gata4*, *Glb1*, and *Cdkn2a* in H_2_O_2_‐induced senescent chondrocytes. Similar to our results obtained in vivo, these factors were increased the in vitro senescence‐induced chondrocytes (Figure S13B, Supporting Information). Furthermore, co‐IP experiments demonstrated that ZMIZ1 was also increased and bound with GATA4 under these conditions (Figure S13C, Supporting Information).

To next test whether GATA4 directly binds to SASP factors, we performed luciferase assays. Sequence analysis revealed that the promoters of *Glb1* and *Cdkn2a* each contain GATA4‐binding sites (‐GATA‐) (Figure S13D, Supporting Information). Our experimental data revealed that overexpression of GATA4 or ZMIZ1 in chondrocytes increased the promoter activities of *Glb1* and *Cdkn2a*, and co‐overexpression of GATA4 and ZMIZ1 synergistically enhanced these promoter activities compared to overexpression of either protein alone (Figure S13E, Supporting Information). This indicates that the ZMIZ1‐GATA4 axis promotes chondrocyte senescence via increasing SASP factor expression. To further investigate the interaction of the ZMIZ1‐GATA4 complex with DNA and map its binding sites, we conducted chromatin immunoprecipitation (ChIP) assays. Our analysis of GATA4‐binding motifs in genomic DNA pulled down by the ZMIZ1 antibody revealed enhancement of the GATA4‐binding motifs located in the promoters of *Mmp3*, *Cox2*, *Glb1*, and *Cdkn2a*, suggesting that these genes are regulated by the ZMIZ1‐GATA4 complex (Figure 4D–H). A heat map generated using the GATA4‐binding motif‐containing fragments from the Ad‐C and Ad‐*Zmiz1* groups revealed that the latter was enriched for GATA4‐binding site‐containing fragments (Figure 4E). Consistent with the results of our heat map analysis, the peak enrichments for *Mmp3*, *Cox2*, *Glb1*, and *Cdkn2a* were significantly stronger in the sequencing data and qRT‐PCR results from Ad‐*Zmiz1* compared to Ad‐C (Figure 4F–H). To assess whether interfering with the ZMIZ1‐GATA4 interaction could inhibit chondrocyte aging, we monitored the expression levels of SASP and catabolic factors in chondrocytes with knockdown of *Gata4*. Our results demonstrated that knockdown of *Gata4* inhibited the expression levels of the ZMIZ1‐GATA4 axis‐induced catabolic factors, *Cdkn2a* and *Glb1*, in Ad‐*Zmiz1* infected chondrocytes (Figure 4I). Together, the above findings demonstrate that ZMIZ1 alone is insufficient to induce chondrocyte aging through SASP expression and suggest that blockade of the ZMIZ1‐GATA4 axis may protect against chondrocyte senescence and cartilage destruction.

### 2.5 K‐7174 Attenuates ZMIZ1‐GATA4 Binding and Reduces the Impacts of the ZMIZ1‐GATA4 Complex

**Figure 5:**
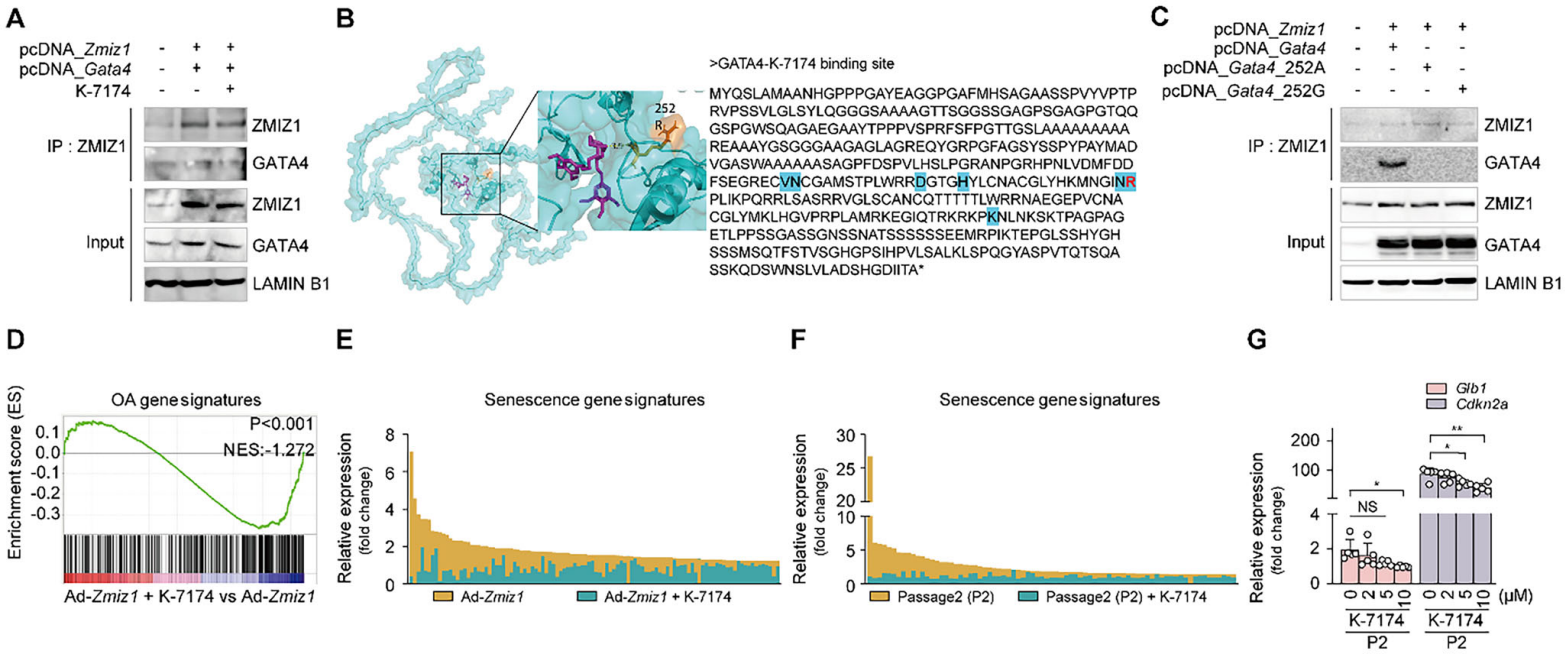
K‐7174 inhibits the ZMIZ1‐GATA4 interaction. (A) Co‐IP analysis performed on chondrocytes co‐transfected with pcDNA‐ZMIZ1 and pcDNA‐GATA4 and treated with K‐7174 (*n* = 3). (B) Computational docking models for GATA4 (cyan) and K‐7174 (magenta) were predicted by Alpha Fold2 and ClusPro 2.0. The binding site of K‐7174, which competes with ZMIZ1, is marked on GATA4 (a.a.: 1–442). (C) Co‐IP of chondrocytes co‐transfected with GATA4‐mutant vectors (*n* = 3). (D) GSEA‐based correlation analysis of OA signature genes in gene sets obtained from chondrocytes infected with Ad‐*Zmiz1* (800 MOI) and treated with or without K‐7174 for 24 h. (E,F) Analysis of senescence gene signature expression pattern in Ad‐*Zmiz1* infected chondrocytes and P2 after K‐7174 treatment. (G) Senescence marker expression changes in passage 2 (P2) chondrocytes treated with K‐7174 (*n* = 5; one‐way ANOVA with Dunnett's multiple comparison test). Data are presented as mean ± SEM for each group. *p < 0.05 and **p< 0.01. NS is non‐significant.

Using in silico prediction and co‐IP analyses, we identified K‐7174 as an inhibitor that can bind at the interaction site between ZMIZ1 and GATA4 and interfere with their complex formation (**Figure** **5**A). Before performing in vitro analysis with K‐7174, we first checked its potential cytotoxicity in human and mouse chondrocytes. Our Lactate dehydrogenase (LDH) assay demonstrated that K‐7174 has no apparent cytotoxicity in normal human or mouse chondrocytes (Figure S14A, Supporting Information). K‐7174 treatment also did not affect the expression levels of catabolic or anabolic factors in chondrocytes (Figure S14B, Supporting Information), nor did it alter ECM (extra‐cellular matrix) synthesis in normal cartilage, as assessed by Alcian blue staining of cultured cartilage explants (Figure S14C, Supporting Information). These data collectively suggested that K‐7174 itself does not exert any undesirable side effect on chondrocytes or cartilage.

To identify the mechanism by which K‐7174 blocks the ZMIZ1‐GATA4 interaction, we predicted the K‐7174‐binding sites of ZMIZ1 and/or GATA4. We found that K‐7174 was predicted to bind multiple amino acids of GATA4, namely 218 V, 219N, 231D, 235H, 251N, 252R, and 323K (Figure 5B). Among them, we noted that the 252R residue of GATA4 is predicted to bind both ZMIZ1 and K‐7174. To demonstrate that this binding site is critical for the ZMIZ1‐GATA4 interaction, we substituted 252R of GATA4 to 252A or 252G and used co‐IP analysis to assess the ZMIZ1‐GATA interaction. Interestingly, both of these single amino acid substitutions decreased the formation of the ZMIZ1‐GATA4 complex (Figure 5C). These results further support our contention that K‐7174 disrupts the ZMIZ1‐GATA4 interaction.

We next tested whether K‐7174 could suppress the ability of ZMIZ1 to up‐regulate catabolic and SASP factors in chondrocytes undergoing both OA pathogenesis and senescence. K‐7174 down‐regulated various *Zmiz1*‐regulated OA signature genes, as assessed by GSEA (Figure 5D). After GSEA, only genes that showed significant changes were isolated and expressed as changes in gene expression in the Ad‐*Zmiz1* group and Ad‐*Zmiz1*+K‐7174 group (Figure 5E). From among the significantly altered genes encoding catabolic factors, we confirmed the decreased expression of several of the encoded proteins (Figure S15A–C, Supporting Information). Moreover, K‐7174‐treated P2 chondrocytes showed down‐regulation of senescence gene signatures and representative SASPs (Figure 5F,G). Given that healthy cartilage is made up of ECM components, such as glycosaminoglycan, proteoglycan, collagen fiber, etc.,^[^ ^39^ ^]^ we examined whether K‐7174 could promote recovery of ECM synthesis and restoration of senescent cartilage through regulation of SASP factor expression. Alcian blue and immunohistological staining showed that K‐7174 protected sulfated proteoglycans and suppressed SASP factor expression in ex vivo‐cultured cartilage explants infected with Ad‐*Zmiz1* (Figure S15D–F, Supporting Information). To test the possibility that K‐7174 might exert its actions mainly by regulating the expression of ZMIZ1 and GATA, rather than inhibiting their interaction, we examined the expression levels of these proteins in IL‐1β‐treated chondrocytes with and without K‐7174 exposure. We found that the transcript levels of *Zmiz1* and *Gata4* were increased in IL‐1β‐treated chondrocytes (Figure S16A, Supporting Information); these changes were not altered by K‐7174, unlike its above‐described effects on the expression levels of *Mmp3*, *Mmp13*, and *Cox2* (Figure S16B, Supporting Information). These results indicate that K‐7174 does not regulate the expression levels of ZMIZ1 and GATA4, but rather competitively inhibits the ZMIZ1‐GATA4 interaction.

### 2.6 Blockade of the ZMIZ1‐GATA4 Axis Inhibits Osteoarthritic Cartilage Destruction and Accelerates the Removal of Senescent Cartilage in OA

**Figure 6:**
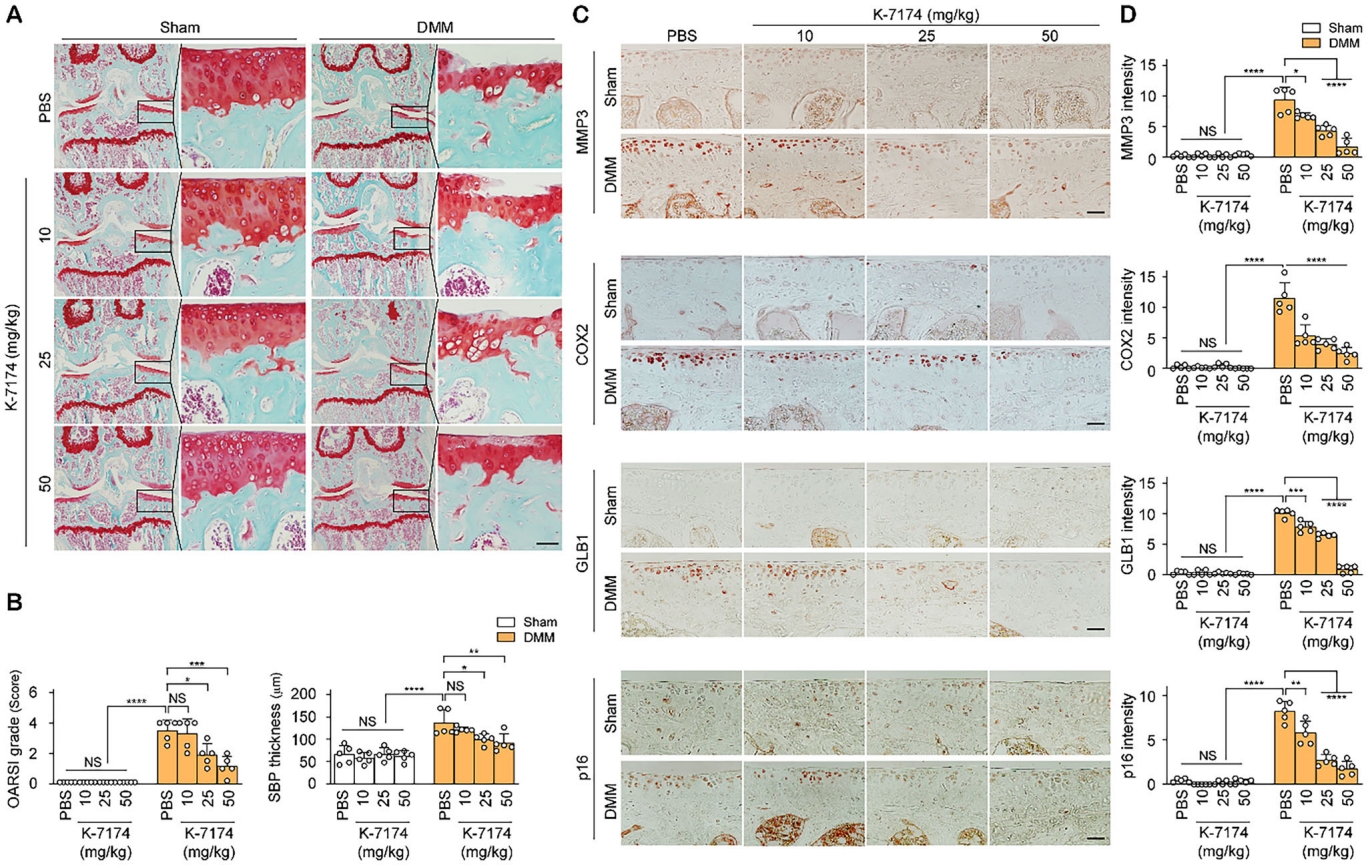
K‐7174 alleviates OA induced by DMM surgery. K‐7174 was orally administered to mice at 4 weeks after DMM surgery. (A) Cartilage tissue staining of mice that received oral administration of K‐7174 at 10, 25, or 50 mg kg^−1^ or PBS (control) (histological image scale bar: 50 µm). (B) Measurement of the extent of OA lesions in mice (*n* = 5; one‐way ANOVA with Dunnett's multiple comparison test). (C,D) Expression levels of MMP3, COX2, and SASP factors in mouse cartilage tissues, as measured by immunohistochemistry and quantified (*n* = 5; scale bar: 50 µm; one‐way ANOVA with Dunnett's multiple comparison test). Magnifications were as follows: Safranin O images, 100 × and 400 × and IHC images, 400 ×. Values are expressed as the mean ± SEM. *p < 0.05, **p < 0.01, ***p < 0.001, and ****p < 0.0001. NS is non‐significant.

To explore the in vivo function of K‐7174, we assessed how different concentrations of K‐7174 impacted DMM‐induced OA in mice (Figure S17A, Supporting Information). Our group previously reported that catabolic factor expression and OA manifestations were gradually increased at 4 weeks after DMM surgery in the same mouse strain used in the present study.^[^ ^29^, ^31^ ^]^ When K‐7174 was orally administered after the onset of OA phenotypes, cartilage destruction diminished as the concentration increased; moreover, there was no evidence of K‐7174‐induced cytotoxicity to the liver, lung, or synovium (**Figure** **6**A,B; Figures S17B and S18, Supporting Information). The inhibitor dose‐dependently decreased the cartilage expression of representative catabolic factors and the OA‐related enhancement of SASP factors, while increasing the expression of COL2A1 (Figure 6C,D; Figure S19A, Supporting Information). To explore a direct effect of K‐7174 on OA development, we additionally performed weekly IA injections of K‐7174 into the knee joints of 60‐week‐old mice, beginning at 4 weeks after DMM surgery (Figure S20A, Supporting Information). Similar to the results obtained upon oral administration of K‐7174, IA injection of K‐7174 into 60‐week‐old mice effectively abrogated cartilage destruction (Figure S20B,C, Supporting Information), suppressed several factors such as MMP3, COX2, GLB1, and p16, and restored COL2A1 expression (Figure S20D,E, Supporting Information). Moreover, although oral administration of K‐7174 blocked cartilage destruction (Figure 6) and recovered COL2A1 expression in DMM‐induced OA mice (Figure S19A, Supporting Information), it did not do so by altering the protein expression levels of ZMIZ1 or GATA4 (Figure S19B,C, Supporting Information). These data collectively suggest that the ZMIZ1‐GATA4 axis is a key regulator of chondrocyte senescence and osteoarthritic cartilage destruction, and further support the idea that K‐7174, which inhibits the ZMIZ1‐GATA4 interaction, could possibly be developed as a potential senolytic drug and OA treatment.

## 3 Discussion

OA can be triggered and promoted by various factors, including aging.^[^ ^15^ ^]^ Healthy cartilage cushions joints and helps them move smoothly and easily, whereas cartilage degeneration makes physically demanding tasks more difficult and taxing on the body.^[^ ^17^ ^]^ Senescent chondrocytes accumulate in cartilage with age and contribute to OA development.^[^ ^9^, ^16^ ^]^ However, it is unclear why aged cartilage exhibits increased susceptibility for cartilage degeneration and OA development via increased collagenase activity and PGE_2_ production leading to ECM degradation.^[^ ^13^, ^17^ ^]^ Given ongoing research showing that cell clearance may be effective in treating certain disease,^[^ ^40^ ^]^ it is thought that removal (clearance) of senescent chondrocytes expected to affect homeostasis and suppress cartilage tissue damage.^[^ ^10^, ^41^, ^42^, ^43^ ^]^ However, the main transcriptional regulators involved in clearing senescent chondrocytes remain unknown. Here, we first show that 60‐week‐old mouse cartilage exhibited increased susceptibility to OA development, along with increased chondrocyte senescence and catabolic factor expression. From among the genes found to be up‐regulated in cartilage at 60 weeks of age, we identified ZMIZ1 as a novel transcriptional co‐factor that appears to contribute to the sensitivity of aged cartilage to OA pathogenic conditions. P16 and senescence‐associated β‐galactosidase (SA‐β‐gal or GLB1), which are senescence‐associated secretory phenotype (SASP) factors,^[^ ^15^, ^16^ ^]^ were increased by overexpression of ZMIZ1. As the SASP factors can contribute to inflammation and tissue destruction,^[^ ^5^, ^44^ ^]^ this suggested that ZMIZ1 could potentially induce OA by promoting chondrocyte senescence. GSEA demonstrated that *Zmiz1* overexpression is significantly related to gene signatures of cellular senescence and OA‐induced catabolic factors. OA cartilage destruction and inflammation are caused primarily by the upregulation of MMP3 and COX2, respectively.^[^ ^29^ ^]^ We found that Ad‐*Zmiz1* infection up‐regulated MMP3 and COX2. Furthermore, we observed that cartilage‐specific *Col2a1*‐*Zmiz1* Tg mice exhibited accelerated cartilage senescence and cartilage destruction, whereas intra‐articular injection of Ad‐sh*Zmiz1* suppressed these parameters. Indeed, ZMIZ1‐overexpressing cartilage exhibited enhancement of OA pathogenesis under triggers such as mechanical stress or exposure to pro‐inflammatory cytokines. Our results collectively suggest that ZMIZ1 accelerates chondrocyte senescence and OA development.

The *Zmiz1* gene encodes several domains whose functions have not been well characterized. Our protein structural homology modeling predicted that ZMIZ1 could bind the transcription factor, GATA4, and our transcriptional analysis suggested that ZMIZ1 can facilitate the function of GATA4. ZMIZ1 has been studied in neurological diseases and leukemia,^[^ ^22^, ^23^ ^]^ and GATA4 is known to be essential for proper development and survival.^[^ ^45^ ^]^ Previous studies found that the regulation of GATA4‐mediated transcription is associated with cellular senescence and rheumatoid arthritis,^[^ ^6^, ^46^, ^47^, ^48^, ^49^ ^]^ but its potential association with OA had not previously been examined. The function of ZMIZ1 in chondrocytes was largely unknown prior to this work: Here, we reveal for the first time that an interaction between ZMIZ1 and GATA4 is essential for the induction of cartilage degeneration and chondrocyte senescence in OA. Our molecular docking analysis suggested that ZMIZ1 can directly bind to a specific sequence of GATA4, whereas K‐7174 could interfere with their interaction at this binding site to inhibit functions downstream of ZMIZ1‐GATA4 binding. Although K‐7174 was previously reported as an anti‐inflammatory agent,^[^ ^50^, ^51^ ^]^ we herein suggest that it may also function as a senolytic drug (one that removes senescent chondrocytes) and thus could potentially be developed for OA treatment. ABT‐263, UBX0101, and Fisetin were previously reported as senolytic drugs and shown to act in this manner by targeting the PI3K/AKT/mTOR, NF‐kB, BCL‐2, and p65 pathways.^[^ ^52^ ^]^ Here, we add K‐7174 as a novel candidate senolytic drug for OA therapy that acts by effectively blocking the ZMIZ1‐GATA4 interaction.

We show that the application of K‐7174 in vitro and in vivo under senescence‐ and OA‐mimicking conditions down‐regulated SASP and catabolic factors and protected against cartilage destruction by inhibiting the degradation of sulfated proteoglycans and promoting the removal of senescent chondrocytes. Therefore, K‐7174, as an inhibitor of the ZMIZ1‐GATA4 interaction, appears to suppress chondrocyte senescence and OA development.

Based on these findings, we propose that ZMIZ1 works as a main transcriptional regulator for accelerating chondrocyte senescence and the ZMIZ1‐GATA4 interaction promotes chondrocyte senescence, leading to OA development. Furthermore, our results suggest that K‐7174 can be used as a senolytic drug to treat OA by clearing senescent chondrocytes and inhibiting catabolic factor expression.

Many studies have been conducted to deliver drugs into the body without losing drug activity using collagen or gels.^[^ ^42^, ^53^, ^54^, ^55^ ^]^ In particular, drug delivery using gel type has been found to be effective for aged or damaged OA cartilage.^[^ ^42^, ^54^, ^55^ ^]^ This delivery system is expected to have potential applications in developing K‐7174 into a more effective senolytic drug.

## 4 Experimental Section

### Human OA Cartilage

Human cartilage samples were obtained from individuals aged 63–80 years undergoing total knee arthroplasty (Table S2, Supporting Information). All patients provided written informed consent, and the collection was approved by the Institutional Review Board of the Catholic University of Korea (UC14CNSI0150). The provided human cartilage tissue was embedded in paraffin and sliced ​​into 5 µm thick slices for experiments.

### Animals and Skeletal Staining

C57BL/6 mice weighing ≈18–20 g were purchased from DBL (Chung‐Cheong Bukdo, South Korea). Cartilage‐specific *Zmiz1* transgenic (*Col2a1*‐*Zmiz1*) mice were generated using the *Col2a1* promoter and enhancer (Macrogen, Seoul, South Korea), as described previously,^[^ ^31^, ^33^, ^56^ ^]^ and were confirmed by genotyping of transgenic (Tg) mice and littermate wild‐type (WT) controls. One‐day‐old Tg and WT mice were used for bone and cartilage staining. Mice were fixed in 95% EtOH for 4 days, treated with acetone for 3 days, and stained with a mixture of Alcian blue (Sigma, Missouri, USA, A9186), Alizarin red (Sigma, A5533), acetic acid (Sigma, 695092), and 95% EtOH for 10 days. Stained samples were then treated with KOH (Sigma, 221473) and de‐stained with glycerol (Junsei, Tokyo, Japan, 27210‐0350). Stereo microscopy (Leica, Wetzlar, Germany) was used for imaging and measurement of skeletal length. All animal experiments were approved by the Animal Care and Use Committee of the University of Sungkyunkwan (SKKUIACUC2024‐03‐35‐1).

### Histological Staining

At the conclusion of experiments, mouse knee joints were obtained by tissue dissociation. The isolated tissue was fixed in 4% paraformaldehyde for 1 day and decalcified for 2 weeks. Demineralized knee joint samples were serially dehydrated with 30, 50, 70, 90, 95, and 100% EtOH, treated with xylene, and embedded in paraffin. Embedded samples were sliced into 5‐µm‐thick sections and Safranin O and immunohistological staining were performed.

For safranin O staining, sectioned samples were sequentially treated with xylene and EtOH (100, 95, 90, 70, 50, and 30%) and stained with hematoxylin (Dako, Glostrup Kommune, Danmark, S3309). Bone and cartilage were than stained with Fast Green (Sigma, F7252), and Safranin O (Sigma, S2255), respectively as described previously.^[^ ^31^ ^]^ For soft tissues (lung and liver), the above‐described steps from ethanol dehydration to paraffin embedding were performed at 1 day after fixation. The tissues were then sectioned, hydrated as described for Safranin O staining, and stained with hematoxylin and eosin (H&Ε; Dako, CS70130‐2).

For immunohistological staining, tissues were deparaffinized, treated with xylene, and hydrate with EtOH. Antigens were retrieved using 0.1% trypsin EDTA, and primary antibodies against proteins of interest (ZMIZ1, Genway, CA, USA, GWB‐MN069F; MMP3, Proteintech, IL, USA, 17873‐1‐AP; COX2, Proteintech, 12375‐1‐AP; p16, Proteintech, 10883‐1‐AP; Glb1, Invitrogen, MA, USA, a‐11132; COL2A1, Chemicon, Land Hessen, Germany, MAB8887; GATA4, Invitrogen, PA1‐102; and p21, Invitrogen, MA5‐14949) were applied overnight. Secondary antibodies (Dako, K4061) were applied and color development was performed using an AEC staining kit (Sigma, AEC101‐1KT).

Safranin‐O‐stained sections were evaluated for cartilage damage by scoring of OARSI grade and subchondral bone plate thickness. Immunohistochemistry results were quantified using the ImageJ software (NIH, USA). Images were converted to 8‐bit and thresholds were measured to compare the expression of each target protein.

### Experimental OA in Mice

OA was induced by DMM surgery as previously described^[^ ^29^, ^57^ ^]^ using 12‐week‐old male WT and *Col2a1*‐ *Zmiz1* Tg mice and 60‐week‐old aged WT mice. Where indicated, intra‐articular injection was performed to introduce Ad‐sh*Zmiz1* (1 × 10^9^ plaque‐forming units; PFU) into to DMM‐operated mouse knee joints. Ad‐Sh*Zmiz1* was injected once a week for 6 weeks, starting 4 weeks after surgery. PFU (rather than MOI) was used for in vivo experiments involving virus infections because quantification of infectious viral particles was crucial for understanding the virus's actual infective potential in a biological system.^[^ ^31^ ^]^ Ad‐sh*Zmiz1* was purchased from Vector Biolabs (USA). Sham‐operated animals and animals injected with Ad‐control (Ad‐C) were used as controls. K‐7174 (MedChemExpress, NJ, USA, HY‐12743) was dissolved in sterilized water at the concentration used for each experiment and administered intra‐articulary and orally. In the injection experiment, each concentration was injected at a maximum volume of 10 µL, and in the oral administration experiment, the concentration was orally administered at a maximum volume of 100 µL. In each experiment, the drug was administered for 6 weeks, starting 4 weeks after DMM surgery, by injection once a week or by oral administration three times a week. PBS was used as a control for K‐7174.

### Chondrocyte and Explant Cultures

Mouse articular chondrocytes were obtained from the femoral condyles and tibial plateaus of 5‐day‐old ICR mice as described previously.^[^ ^29^, ^58^ ^]^ The isolated cartilage was treated with collagenase to separate cells, and 2.75 × 10^5^ cells were seeded to 35‐mm cell culture dishes and incubated at 37 °C in Dulbecco's modified Eagle's medium (DMEM) supplemented with 10% fetal bovine serum (FBS) and antibiotics. Normal human chondrocytes were purchased from Cell Application (CA, USA, 402‐05a) and cultured in DMEM supplemented with 10% FBS and antibiotics at 37 °C in a 5% CO_2_ incubator. The medium was replaced every 2 days until the cells grew to 95% of confluence and cytotoxicity was examined in cultures treated with 0, 2, 5, or 10 µm of K‐7174 (MedChemExpress, HY‐12743A) for 24 h before harvest.

Femoral and tibial cartilage explants were also isolated from the femoral condyles and tibial plateaus of 5‐day‐old ICR mice. Isolated explants were cultured in DMEM containing 10% FBS, infected with or without Ad‐*Zmiz1* infection, and treated with 0, 2, 5 or 10 µm of K‐7174 for 72 h, during which time the DMEM, Ad‐*Zmiz1*, and K‐7174 were refreshed daily. After the explant infection experiment, all explants were washing with PBS then fixed with 4% paraformaldehyde, dehydrated with EtOH, embedded in paraffin, and sectioned at 5‐µm thickness. Proteoglycan restoration within the excised tissues was assessed by Alcian blue staining (pH 2.5). After removing paraffin and rehydrating, the sections were stained with Alcian blue (Sigma, A5268) dissolved in 0.1 N HCl.

### Cellular Senescence

To induce chondrocyte senescence, cultured primary chondrocytes (passage 0, P0) were subcultured until P2. When cells reached confluence on a 35‐mm cell culture dish, they were detached and counted, and half of the cells were subcultured until P2. The P1 cell medium was changed every other day for 6 days, whereupon the cells were subcultured to passage 2 (P2) cells. P2 chondrocytes were harvested, and β‐gal staining was performed according to the manufacturer's recommendations (Cell Signaling, MA, USA, 9860S). Further passages with medium replacement every other day were used to obtain P5 chondrocytes. As another way to induce cell senescence, cells were treated with 200 µm H_2_O_2_ for 24 h 1 day before harvest. P2, P5, and H_2_0_2_‐treated cells were processed for RNA and co‐IP analyses. For histological staining of senescent cartilage, SA‐β‐gal was not detected because its enzymatic activity renders it undetectable in formalin‐fixed paraffin‐embedded (FFPE) tissues.^[^ ^59^ ^]^ Instead, a primary antibody against GLB1 (Invitrogen, a‐11132) was used, which was a minor product of the gene encoding β‐gal.

### Cell Treatment, Overexpression, and Knockdown

The pro‐inflammatory cytokines, IL‐1β (GenScript, NJ, USA, Z02922), TNF‐α (GenScript, Z01001), and IL‐17 (GenScript, Z02970), were dissolved in sterilized water. Mouse primary chondrocytes were treated with IL‐1β (1 ng mL^−1^), TNF‐α (50 ng mL^−1^), or IL‐17 (50 ng mL^−1^) at the indicated time points before cell harvest. Ad‐C (Control) and Ad‐*Zmiz1* were purchased from Vector Biolabs. Primary mouse chondrocytes were cultured for 3 days and then infected with adenovirus encoding *Zmiz1* (Ad‐*Zmiz1*) at the indicated multiplicity of infection (MOI); the medium was changed at 2 h post‐infection and cells were incubated for 24 h post‐infection. MOI was used as the unit of measure for the in vitro work because measuring the ratio of infectious viral particles to the number of target cells works well in controlled environments where the number of target cells was well‐defined (e.g., cell cultures).^[^ ^29^ ^]^ K‐7174 was applied to Ad‐*Zmiz1*‐infected chondrocytes for the same period of time. *Zmiz1* and *Gata4* siRNA were transfected into chondrocytes for 48 h using RNAiMAX (Invitrogen, 13778‐150).

### Micro‐CT Analysis

Mouse samples were scanned using a SkyScan 1173 (Bruker, Kontich, Belgium) and the following key scan parameters: source voltage, 90 kV; source current, 88 µA; isotropic image voxel size, 10 µm; exposure, 500 ms; frame average, 4; number of projections, 800; rotation step, 0.3 degree; and scan, 180‐degrees. Mouse bone mineral density was scanned and acquired projections were reconstructed in NRexon (Bruker) using the following reconstruction parameters: ring artifact correction, 7; beam hardening correction, 40%; attenuation coefficient dynamic range, 0–0.034; and filter, A1 1.0 mm. Dataviewer (Bruker) and CTAn (Bruker) were used to reorient the reconstructed cross‐sections. A volume of interest (VOI) was defined as follows: ≈0.5 mm proximal to the growth plate and spanning 1.6 mm in length and width. The VOI analysis for subchondral bone was performed as previously described.^[^ ^29^ ^]^ Trabecular bone volume (BV/TV), trabecular thickness (Tb. Th), trabecular number (Tb. N), and cortical thickness (Ct. Th) were calculated. Bone objects were segmented using a global threshold (81‐255) rather than a dynamic threshold. Despeckle plugin (Billerica, MA, USA) was used for custom processing. CTvox (Bruker) was used to create 3D images of old and young mouse samples and visually represent the trabecular thickness.

### Bioindentation

The cartilage elasticities of mouse femoral and tibial cartilage were measured with an Anton Paar Bioindenter (Anton Paar, Graz, Austria) and a ruby ball indenter (200 µm). All cartilage tissue was freshly isolated and stored in PBS prior to measurement, and the tissues were fixed on slides. After specifying at least four different locations in the cartilage area to measure elasticity, the elasticity of the tissues was measured using a ruby ​​ball. The elasticity of cartilage was measured through the calculated ErHz [MPa] and the force‐indentation depth‐loading curve was drawn.

### Gene Set Analysis (IPA and GSEA)

To identify transcriptional regulators (or another mRNA subset) that differed between old and young cartilage, total RNA was isolated from cartilage samples obtained from 12‐ and 60‐week‐old mice and analyzed using an Affymetrix GeneChip array (Affymetrix Mouse Gene 2.0 ST array) and the Affymetrix protocol (both from Macrogen, Seoul, Korea).

Besides this, various gene sets were assessed, including those representing Ad*‐Zmiz1* infection, passaged chondrocytes, and K‐7174‐treatment groups for each condition. Various analyses were performed with the gene sequence sets obtained in this way. In particular, after performing GSEA analysis on the Ad‐*Zmiz1*+ K‐7174 gene set together with the senescence signatures, a separate list of genes that showed significant change values in the GSEA analysis was obtained. The actual expression values ​​of genes that showed changes in each gene set of Ad‐*Zmiz1* and Ad‐*Zmiz1*+K‐7174 were displayed graphically using Prism 8.4.3 (GraphPad, USA). This shows that significant genes in each signature were downregulated by K‐7174. Inhere total RNA was isolated from each group and gene sets were obtained through RNA‐sequencing using the Illumina Novaseq6000 platform (DNA LINK, Seoul, Korea).

The transcription factor gene set of the IPA software was used to classify the transcriptional regulators of the 12‐ and 60‐week‐old mouse gene sets. To characterize the possible relationship with OA, GSEA was used to compare a given gene set with a previously reported OA gene signature.^[^ ^60^ ^]^ Likewise, the senescence gene set of the IPA software was compared with the gene sets, and senescence was confirmed through GSEA and direct analysis of gene expression levels.

### Transcription Factor Analysis

Transcription factor array analyses were performed with a Cignal 45‐Pathway Reporter Array (Qiagen, Hilden, Germany). Primary chondrocytes were seeded to 96‐well plates and treated with hyaluronidase for 4 h to enhance the transfection efficiency. Stabilized cells were transfected with reporter constructs using Attractene (Qiagen, 301005). Transfected cells were infected with Ad‐C or Ad‐*Zmiz1* for 2 h. After 36 h, firefly luciferase and Renilla luciferase were measured using a Dual‐Glo Luciferase Assay System (Promega, WI, USA, E2920). To normalize the transfection efficiency, firefly luciferase was quantified relative to Renilla luciferase.

### Immunoprecipitation

The interaction of the endogenous ZMIZ1 and GATA4 proteins was assessed in chondrocytes treated with IL‐1β (1 ng mL^−1^) or H_2_O_2_ (200 µm) for 24 h. The treated cells were detached and proteins were extracted using an NE‐PER (Nuclear and Cytoplasmic Extraction Reagents) kit (Thermo Scientific, MA, USA, 78835) according to the manufacturer's recommendations. For co‐IP using a plasmid vector, sequences encoding *Zmiz1* (accession ID: NM_183208.4) and *Gata4* (accession ID: NM_001310610.1) were cloned into pcDNA3.1 (BioD, Gwangmyeong‐si, Korea). K‐7174 binding‐site mutants of *Gata4* (pcDNA3.1_*Gata4* 252A and pcDNA3.1_ *Gata4* 252G) were generated from the synthesized *Gata4* cDNA and subcloned (BioD). These vectors were transfected at 2.5 µg of vector per 100 mm cell culture dish using PolyJet (SignaGen Laboratories, Shandong Province, China) as appropriate for each experiment. Cytoplasmic and nuclear proteins were isolated using an NE‐PER kit, and nuclear proteins were used as inputs or for IP. Proteins were quantified, an equal amount of ZMIZ1 antibody (Cell Signaling, 89500) was added, and samples were mixed overnight at 4 °C. Protein A‐agarose beads (Roche, Basel, Swiss, 11719408001) were added and samples were mixed by rotation overnight at 4 °C. The beads were washed one day later with immunoprecipitation buffer for five times (150 mm NaCl, 1% NP‐40, 50 mm Tris, and 5 mm NaF), and the proteins were separated from the beads by heating and resolved by SDS‐PAGE. Antibodies were used against the following: ZMIZ1 (Cell Signaling, 89500), GATA4 (Santa Cruz, TX, USA, SC‐25310), and LAMIN B1 (loading control; Abcam, ab1648).

### Promoter Gene Assay

To perform gene promoter assays, GATA4‐binding motifs (‐GATA‐) were first identified in the promoter regions of *Glb1* and *Cdkn2a*. The relevant promoter gene segments were inserted into the pGL3‐basic plasmid vector and cloned (BioD). pcDNA3.1‐*Zmiz1* and pcDNA3.1‐*Gata4* plasmids were cloned using *Zmiz1* (NM_183208.4) and *Gata4* (NM_001310610.1) cDNAs (BioD). These vectors were co‐transfected at 300 ng per 35 mm cell culture dish along with a vector encoding β‐gal (150 ng per 35 mm cell culture dish) into chondrocytes using PolyJet (SignaGen Laboratories). At 48 h post‐transfection, enzyme activities were measured in cell extracts using a luciferase assay kit (Promega, E1501) and a β‐galactosidase assay kit (Promega, E2000) according to the manufacturer's recommendations. Luciferase activity was normalized to β‐galactosidase activity.

### ChIP (Chromatin Immunoprecipitation)

To further explore the ZMIZ1‐GATA4 interaction, ChIP experiments were performed. Chondrocytes infected with Ad‐C and Ad‐*Zmiz1* were cross‐linked with 37% formaldehyde, and 8 × 10^6^ chondrocytes were collected and sheared by sonication (12% amplitude, 9 sec/9 sec on/off for 10 min). Genomic DNA (gDNA) was obtained and subjected to IP using a ZMIZ1 antibody (Cell Signaling, 89500) and the eluted gDNA fragments were subjected to qRT‐PCR and sequencing. Primers (Table S3, Supporting Information) were designed to flank the GATA4‐binding sites in the promoter regions of *Mmp3*, *Cox2*, *Glb1*, and *Cdkn2a*, and transcript levels in Ad‐C and Ad‐*Zmiz1* groups were compared through qRT‐PCR.

Raw paired‐end FASTQ data were processed with cutadapt (ver. 1.1.8)^[^ ^61^ ^]^ to remove adaptors and low‐quality reads (cut‐off Q < 30). The filtered reads were aligned to the *Mus musculus* (mm10) genome using bowtie2 (ver. 2.5.4) and reads with MAPQ value < 30 were discarded using SAMtools (ver. 1.9).^[^ ^62^ ^]^ Duplicate reads were identified and removed using Picard (ver. 2.27.5) (http://broadinstitute.github.io/picard/). The mapped BAM files were converted to the BigWig format using bamCoverage, with reads per genome coverage (RPGC) normalization applied via deepTools (ver. 3.5.5).^[^ ^63^ ^]^ Peak calling was performed using MACS3 (ver. 3.0.1)^[^ ^64^ ^]^ with a *p‐*value threshold of ≤ 0.001; peak regions that overlapped with ENCODE blacklist regions were excluded.^[^ ^65^ ^]^ The MEME suite was used to evaluate the signal intensity of the GATA4 motif within the peaks of each sample.^[^ ^66^ ^]^ FIMO was employed to search for motif occurrences, using the GATA4 motif file downloaded from the JASPAR database.^[^ ^67^ ^]^ Heat maps were generated using deepTools, and the genome tracks were visualized using Integrative Genomics Viewer (IGV) (ver. 2.17.4) with RPGC‐normalized BigWig files.

### PGE_2_ and Collagenase Activity Assays

Mouse primary chondrocytes were seeded and treated with Ad‐*Zmiz1* or K‐7174, and inflammation‐induced secretion of PGE_2_ to the cell supernatant was measured using a PGE_2_ immunoassay kit (R&D Systems, MN, USA, KGE004B) according to the manufacturer's protocol. To assess collagenase activity, chondrocyte cultures were subjected to Ad‐*Zmiz1* infection or K‐7174 treatment, and conditioned media were processed with an EnzChek Gelatinase/Collagenase Assay Kit (Invitrogen, e12055). A portion of the cell supernatant from each experiment was used for this experiment. Activity was measured using a VICTOR X3 microplate reader (PerkinElmer, Waltham, MA, USA) at EX/EM = 480/530 nm.

Reverse transcription‐polymerase chain reaction (RT‐PCR), quantitative RT‐PCR (qRT‐PCR), and Western blot assay:

Total RNA from primary cultured articular chondrocytes was reverse‐transcribed and used to generate cDNA. The utilized PCR primers and experimental conditions are summarized in Table S3 (Supporting Information). Transcript levels were quantified by qRT‐PCR (StepOnePlus Real‐Time PCR System; ABI, MA, USA). To analyze protein expression in mouse articular chondrocytes, total cell lysates were prepared in lysis buffer (150 mm NaCl, 1% NP‐40, 50 mm Tris, 5 mm NaF, and 10% SDS) containing 10% sodium deoxycholate and a cocktail of protease inhibitors (Roche, 25178620). The following antibodies were used for Western blot analysis: anti‐ZMIZ1 (Genway, GWB‐MN069F), mouse anti‐ERK (Becton Dickinson, 61048), rabbit anti‐COX2 (Abcam, Cambridge, UK, ab52237), anti‐MMP3 (Abcam, ab52915), anti‐COL2A1 (Chemicon, MAB8887), anti‐aggrecan (Invitrogen, PA1‐1746), anti‐SOX9 (Santa Cruz, SC‐20095), and anti‐p16 (Proteintech, 10883‐1‐AP). ERK was detected as a loading control. MMP3 was isolated from chondrocyte‐conditioned media by trichloroacetic acid and acetone precipitation, as described previously.^[^ ^32^, ^58^ ^]^ The precipitated protein was dissolved in lysis buffer and subjected to SDS‐PAGE. Densitometric analyses were used to quantify band intensities, as applied using AlphaEase FC 4.0 (Alpha Innotech, USA).

### Protein Structural Homology Modeling and Binding Affinity Assay

Homology‐based structural modeling of ZMIZ1 (accession ID: NP_065071.1) and GATA4 (accession ID: NP_001295022.1) was conducted using AlphaFold2 (ver. 2.3.2).^[^ ^68^ ^]^ Computational docking simulations were conducted with AlphaFold2 and ClusPro 2.0 (hydrophobic‐favored scoring scheme).^[^ ^69^ ^]^ The ClusPro scores for the docking model were −1048.5 for the center and −1226.1 for the lowest energy region. Molecular docking analyses were performed using AutoDock Vina (ver. 1.1.2), which was widely used for protein‐ligand docking. All dockings were conducted using the parameters of rigid receptors with fully flexible ligands. The receptor coordinates and docking parameters were prepared using AutoDock MGLTools (ver. 1.5.6).^[^ ^70^ ^]^ Binding affinities for those ligands were evaluated by negative Gibbs free energy (ΔG) scores (kcal mol^−1^). The graphical representation of docking structures was constructed using PyMOL (ver. 1.3; DeLano Scientific, San Carlos, CA, USA).

### Statistical Analysis

Values are presented as the mean ± SEM. Statistically significant differences were evaluated using one‐way ANOVA with Dunnett's multiple comparison test, one‐way ANOVA with Tukey's post‐hoc test, two‐tailed *t*‐test, or a non‐parametric statistical method such as the Mann–Whitney U test.^[^ ^29^, ^31^, ^33^, ^60^ ^]^ Statistical significance was determined at *p* < 0.05. Statistical analyses were performed using the GraphPad Prism 8. 4. 3 software (GraphPad). All experiments were independently performed over at least three replicates. OARSI grade and SBP scoring were quantified according to an ordinal grading system and statistically analyzed using the statistical methods mentioned above.

## Conflict of Interest

The authors declare no conflict of interest.

## Author Contributions

J.N. designed and conducted most of the in vitro and in vivo experiments and wrote the manuscript. H.W., J.Y., and S.I.E. carried out computational analyses of microarrays, GEO, ChIP, and protein structural homology modeling. S.J.K. obtained and evaluated human joint samples. K.P.L. conducted the µCT analysis. J.H.Y. and T.J.P. supported K‐7174‐related in vitro and in vivo analysis. S.I.E. (as co‐corresponding author) and S.Y. conceived, planned, and oversaw the study.

## Supporting information



Supporting Information

## Data Availability

The data that support the findings of this study are available in the supplementary material of this article.
