# Supplementary material for: Blockade of ZMIZ1‐GATA4 Axis Regulation Restores Youthfulness to Aged Cartilage
Source: Adv Sci (Weinh). 2025 Mar 5;12(16):2404311. doi: 10.1002/advs.202404311 (PMC12021034; doi:10.1002/advs.202404311)
Supplement: Supplementary file 1 — Supporting Information [file ADVS-12-2404311-s001.pdf]

ADVANCED SCIENCE
Open Access

Supporting Information

for *Adv. Sci.*, DOI 10.1002/advs.202404311

Blockade of ZMIZ1-GATA4 Axis Regulation Restores Youthfulness to Aged Cartilage

Jiho Nam, Hyunmin Woo, Jihye Yang, Seok Jung Kim, Kwang Pyo Lee, Ji Hoon Yu, Tae Joo Park, Seong-il Eyun and Siyoung Yang**

Supporting Information

Blockade of ZMIZ1-GATA4 axis regulation restores youthfulness to aged cartilage

Jiho Nam, Hyunmin Woo, Jihye Yang, Seok Jung Kim, Kwang Pyo Lee, Ji Hoon Yu, Tae Joo Park, Seong il Eyun, Siyoung Yang**

This supporting information includes **Fig. S1-20** and **Table S1-3.**

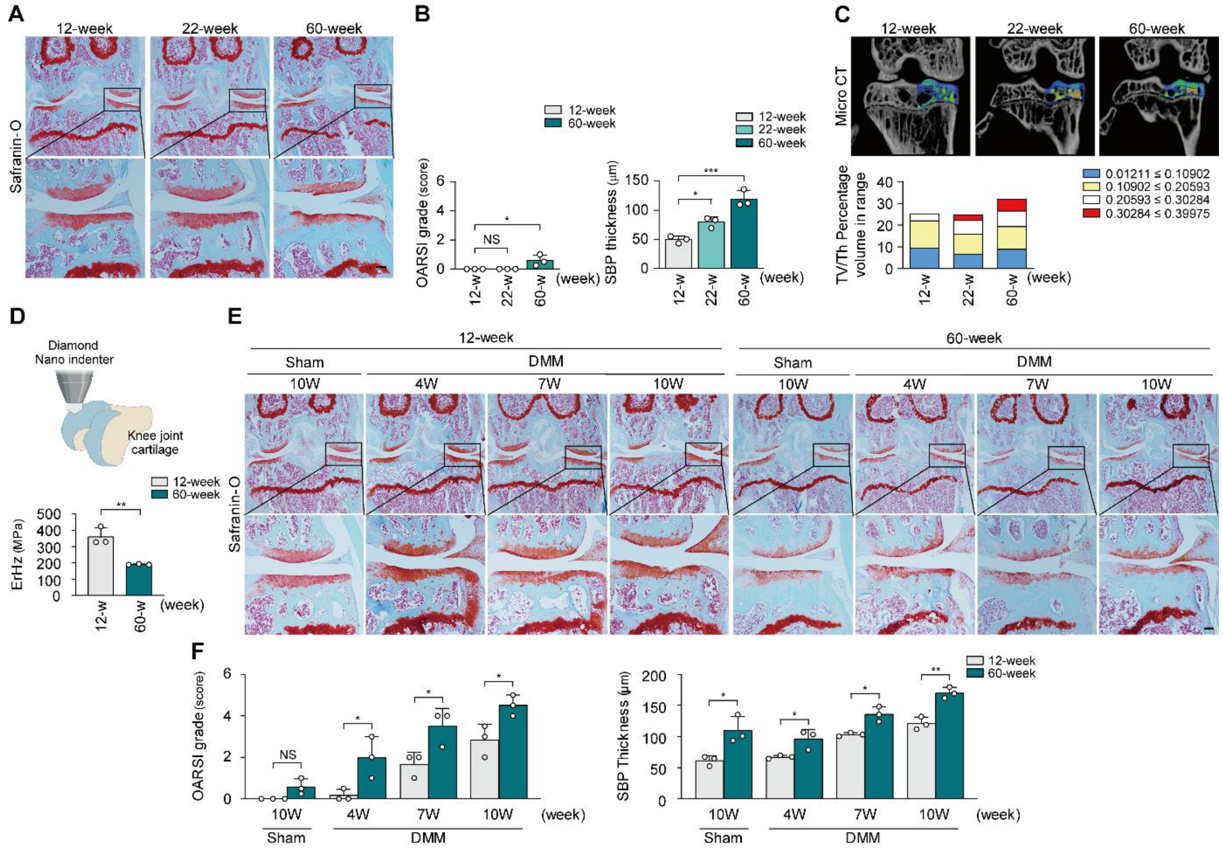

Fig. S1 Aging induces osteoarthritis in mouse knee joints.

(A and B) Cartilage from 12-, 22-, and 60-week-old mice was assessed by Safranin O staining **(A)** (*n* = 3; scale bar: 100 μm) and OA scoring values, OARSI grade, and subchondral bone plate thickness **(B)** (*n* = 3; one-way ANOVA with Dunnett's multiple comparison test). **(C)** Micro-CT analysis of 12-, 22-, and 60-week-old mouse knee joints (*n* = 3). Red color indicates trabecular bone and green indicates cortical bone (upper). Graph showing TV/Th percentage volume in ranges (lower). **(D)** The elastic modulus of cartilage in 12- and 60-week mouse groups, as measured by bioindenter (*n* = 3; two-tailed t-test). **(E)** Cartilage tissue staining of samples obtained at 4-, 7- and 10-weeks post-surgery from young and old mice (*n* = 3; scale bar: 100 μm). **(F)** Measurement of OA lesions in mice of each group (*n* = 3; two-tailed t-test). Safranin O images were taken at magnifications of 40× and 100×. Values are expressed as the mean ± SEM. * $P< 0.05$, ** $P< 0.01$ and *** $P< 0.001$. NS is non-significant.

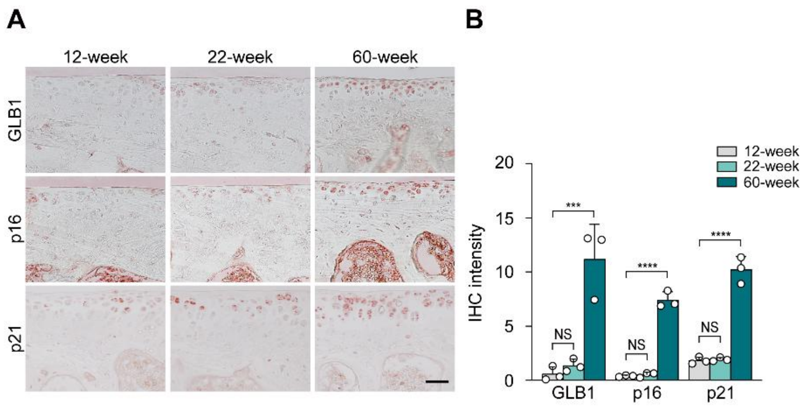

Fig. S2 Expression of GLB1, p16, and p21 in cartilage of young and old mice.
(**A**) Expression of the aging markers, GBL1, p16, and p21, in cartilage samples obtained from 12-, 22- and 60-week-old mice (*n* = 3; scale bar: 50 μm). (**B**) Quantified expression of each factor (*n* = 3; one-way ANOVA with Dunnett's multiple comparison test). IHC images were taken at a magnification of 400×. Values are expressed as the mean ± SEM. *** $P< 0.001$ and **** $P< 0.0001$. NS is non-significant.

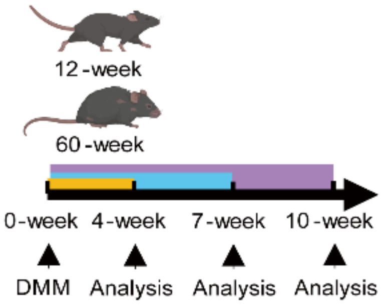

Fig. S3 Workflow for *in vivo* experiments.

Schedule for DMM surgery in 12- and 60-week-old mice. Cartilage sampling and analysis were conducted at 4-, 7-, and 10-weeks post-surgery.

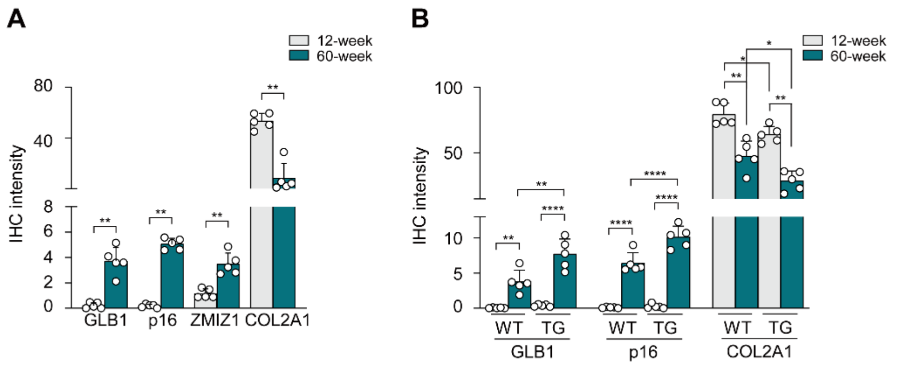

Fig. S4 Quantification of immunohistochemistry results.

(A) Quantification of GLB1, p16, ZMIZ1, and COL2A1 protein expression levels in cartilage tissues from young and old mice (*n* = 5, by Mann-Whitney U test). **(B)** Quantification of GLB1, p16, and COL2A1 in cartilage tissues from young and old WT and *Col2a1-Zmiz1* Tg mice (*n* = 5; Mann-Whitney U test). Values are expressed as the mean ± SEM. * *P*< 0.05, ** *P*< 0.01 and **** *P*< 0.0001.

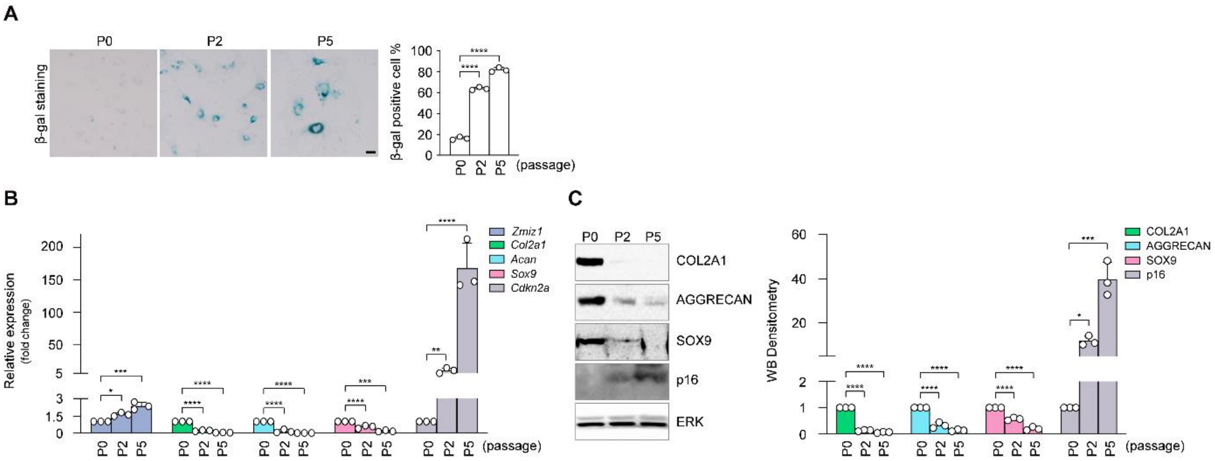

Fig. S5 Expression levels of anabolic and SASP factors in subcultured chondrocytes.

Primary culture chondrocytes (p0) were subcultured to passage 5 (P5), and β-galactosidase staining was performed and gene and protein expression levels were determined in P0, P2, and P5 chondrocytes. **(A)** β-galactosidase staining for chondrocytes of each passage (n = 3; one-way ANOVA with Dunnett's multiple comparison test). **(B)** qRT-PCR analysis of the transcript levels of *Zmiz1*, cartilage anabolic factors (*Col2a1*, *Acan*, *Sox9*) and an aging marker (*Cdkn2a*) in P0, P2, and P5 chondrocytes (n = 3, by one-way ANOVA with Dunnett's multiple comparison test). **(C)** Protein expression of anabolic and aging factors in chondrocytes of each passage, as assessed using WB (left panel) and quantification (right panel) (n = 3; one-way ANOVA with Dunnett's multiple comparison test). β-galactosidase staining images were taken at a magnification of 100×; scale bar: 100 μm. Values are expressed as the mean ± SEM. * P< 0.05, ** P< 0.01, *** P< 0.001 and **** P< 0.0001.

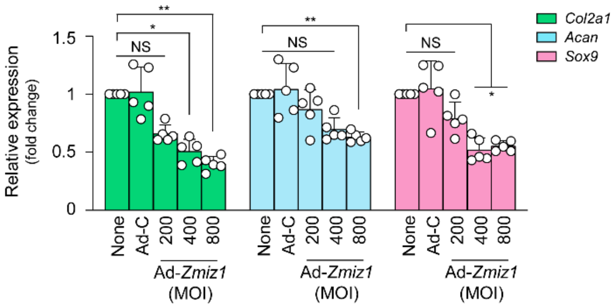

Fig. S6 Ad-*Zmiz1* infection-induced expression of anabolic factors in chondrocytes.
Analysis of anabolic factor (*Col2a1*, *Aggrecan*, and *Sox9*) expression in chondrocytes with Ad-*Zmiz1*-induced overexpression of *Zmiz1* (n = 5; one-way ANOVA with Dunnett's multiple comparison test). Values are expressed as the mean ± SEM. * $P< 0.05$, ** $P< 0.01$ and *** $P< 0.001$. NS is non-significant.

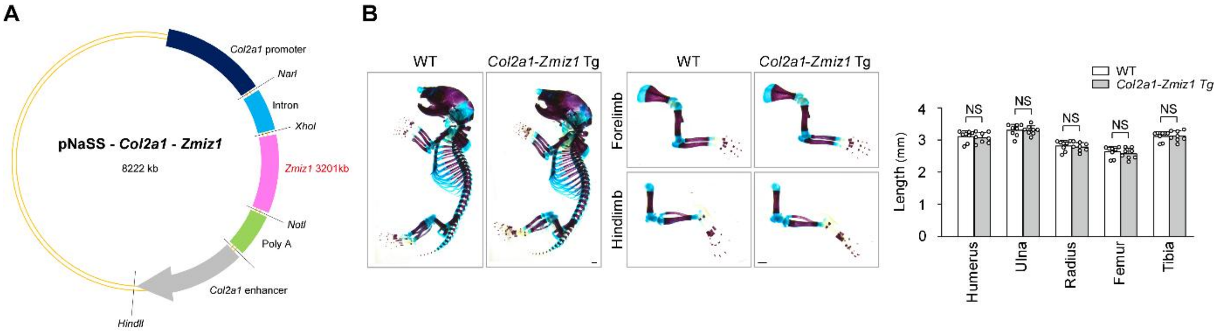

Fig. S7 Generation and characterization of cartilage-specific *Col2a1-Zmiz1* Tg mice.

(A) Vector used to generate cartilage-specific *Zmiz1* Tg mice. **(B)** Skeletal staining comparing skeletal formation between Tg and WT mice (left), and the lengths of forelimbs and hindlimbs (right) (n = 8; Mann-Whitney U test). Values are expressed as the mean ± SEM. NS is non-significant.

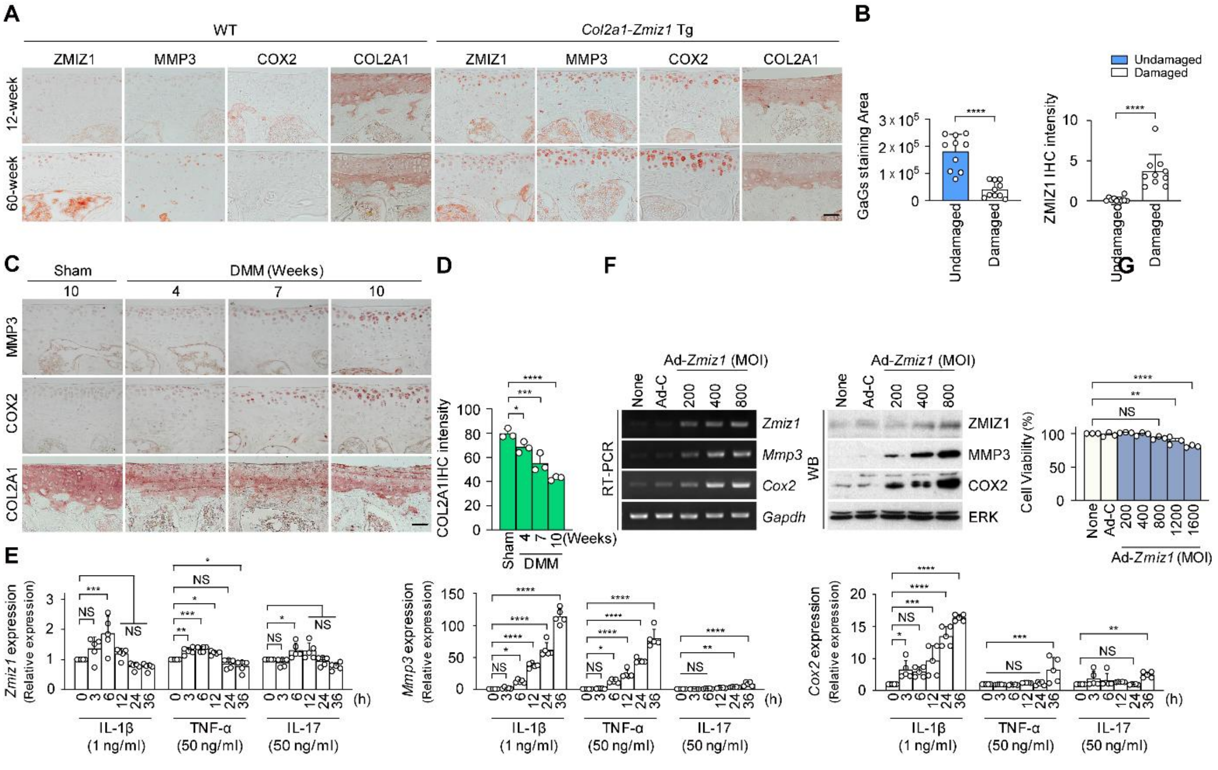

Fig. S8 Characterization of ZMIZ1 overexpression in mouse tissues and cells.

(A) Cartilage tissues of *Col2a1-Zmiz1* Tg mice and WT mice were compared. ZMIZ1, MMP3, COX2, and COL2A1 proteins were detected in mice of each genotype and age (*n* = 5; scale bar: 50 μm). **(B)** Quantification of GAG regions identified through Alcian blue staining and ZMIZ1 expression in human damaged OA cartilage tissues (*n* = 10, by Mann-Whitney U test). **(C)** Expression levels of MMP3, COX2, and COL2A1 in mouse cartilage samples obtained at the indicated weeks after DMM surgery (*n* = 3; scale bar: 50 μm). **(D)** COl2A1 IHC quantification (*n* = 3; one-way ANOVA with Dunnett's multiple comparison test). **(E)** qRT-PCR analysis-based quantification of *Zmiz1*, *Mmp3*, and *Cox2* expression levels in chondrocytes treated with IL-1β (1 ng/ml), TNF-α (50 ng/ml), or IL-17 (50 ng/ml) for the indicated durations (*n* = 5; one-way ANOVA with Dunnett's multiple comparison test). **(F)** ZMIZ1 overexpression was induced by infecting chondrocytes with the indicated MOI of Ad-*Zmiz1* for 36 hours. The transcript and protein levels of each target in mouse articular chondrocytes were measured by RT-PCR (left) and WB (right), respectively (*n* = 5). **(G)** Cell viability of cultures infected with the indicated MOI of Ad-*Zmiz1* (*n* = 3; one-way ANOVA with Dunnett's multiple comparison test). IHC images were taken at a magnification of 400×. Values are expressed as the mean ± SEM. * *P*< 0.05, ** *P*< 0.01, *** *P*< 0.001 and **** *P*< 0.0001. NS is non-significant.

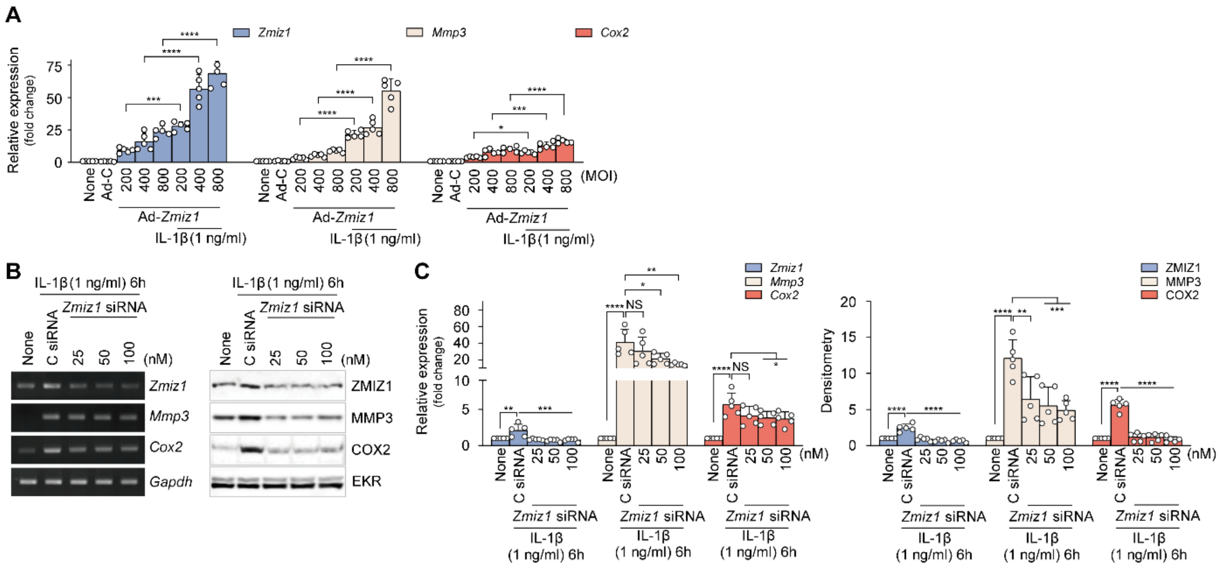

Fig. S9 ZMIZ1 regulates OA pathogenic factors.

(A) qRT-PCR-based quantification of *Zmiz1*, *Mmp3*, and *Cox2* in Ad-*Zmiz1*-infected chondrocytes treated with IL-1β (n = 5; one-way ANOVA and Tukey's post-hoc test). **(B** and **C)** Mouse chondrocytes treated with IL-1β (1 ng/ml) to mimic OA were subjected to siRNA-mediated knockdown of *Zmiz1*. **(B)** Expression of catabolic factors under *Zmiz1* knockdown was assessed at the transcript (left) and protein (right) levels (n = 5 each). **(C)** These expression levels were quantified by qRT-PCR (left) and densitometry (right). Statistical analyses were conducted by one-way ANOVA with Dunnett's multiple comparison test. Values are expressed as the mean ± SEM. * P< 0.05, ** P< 0.01, *** P< 0.001 and **** P< 0.0001. NS is non-significant.

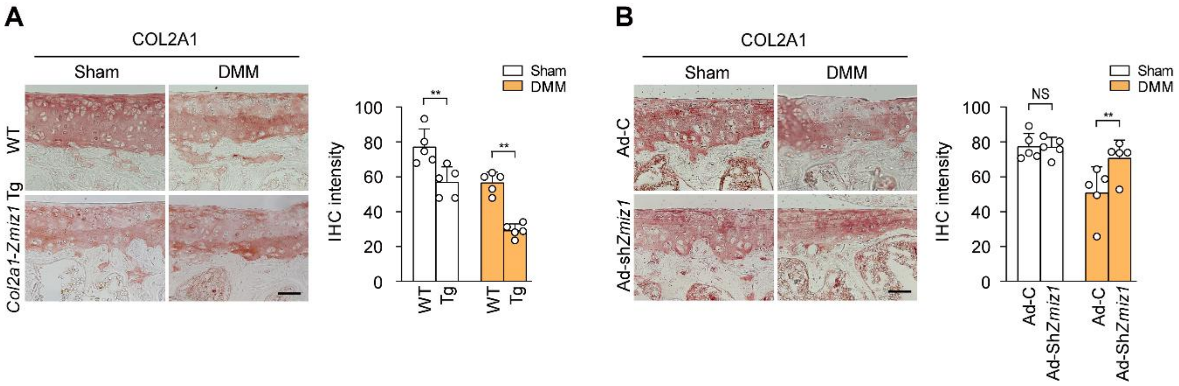

Fig. S10 ZMIZ1 regulates COL2A1 expression in cartilage.

(A) Confirmation and quantification of COl2A1 expression in *Col2a1-Zmiz1* Tg and WT mice (*n* = 5; Mann-Whitney U test). **(B)** Changes in COL2A1 expression and quantification under ZMIZ1 knockdown (*n* = 5; Mann-Whitney U test). Histological image scale bar: 50 μm. All images were taken at a magnification of 400×. Values are expressed as the mean ± SEM. ** $P < 0.01$ and NS is non-significant.

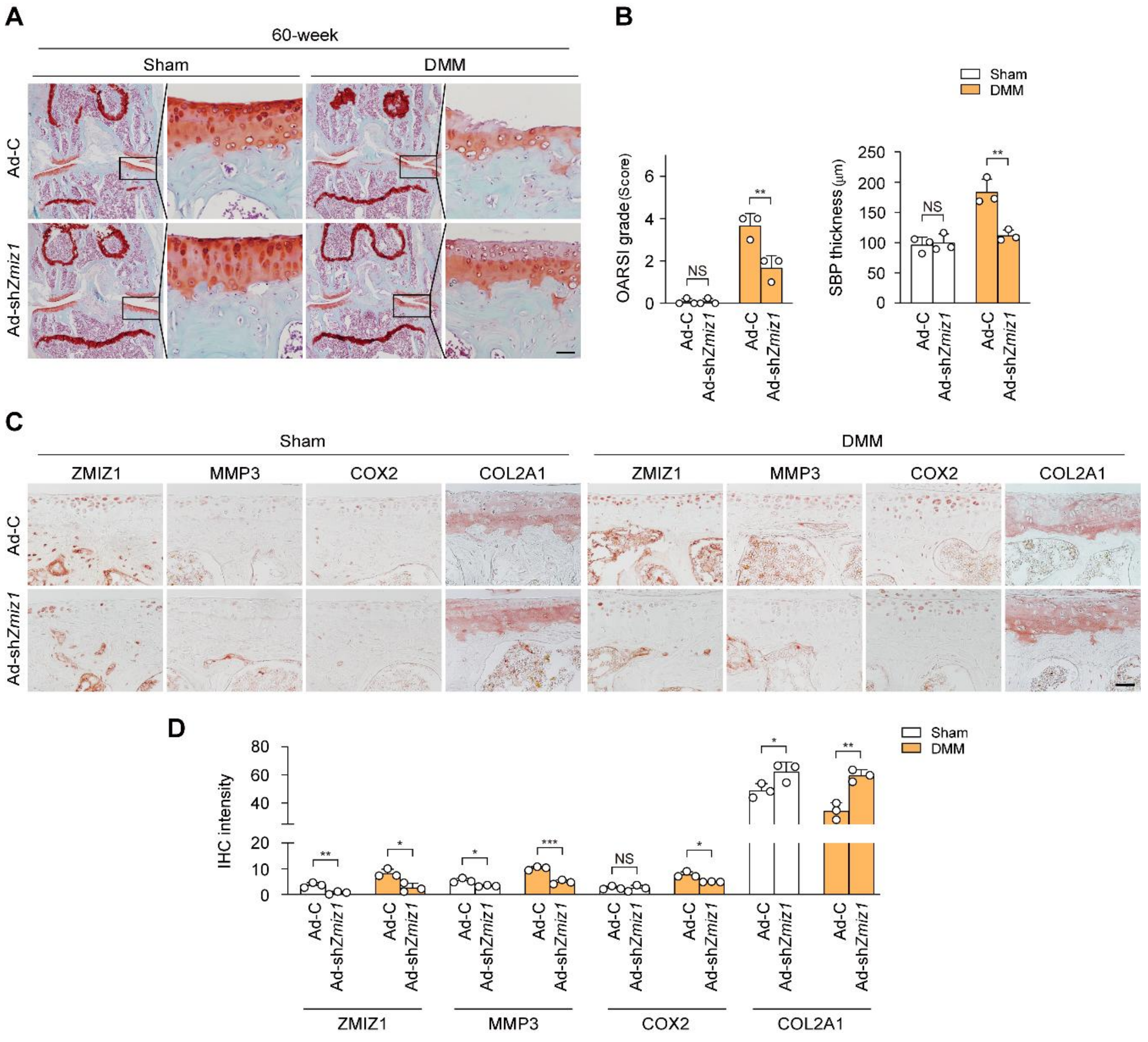

Fig. S11 Ad-*Zmiz1* shRNA injection recovers degenerated cartilage in aged mice.

The effect of ZMIZ1 inhibition on DMM-induced cartilage damage in 60-week-old mice. At 4 weeks after DMM surgery, *Zmiz1* shRNA (Ad-sh*Zmiz1*) was injected into mouse knee joint once a week for a total of 6 weeks. **(A)** Safranin O staining of injected cartilage (n = 3). **(B)** OARSI grade and SBP thickness measurement (n = 3; Two tailed t-test). **(C** and **D)** Immunohistochemical staining and quantification of ZMIZ1, MMP3, COX2, and COL2A1 in Ad-sh*Zmiz1*-injected 60-week-old mice (n = 3; Two tailed t-test). Safranin O staining and IHC image scale bars: 50 µm. Images were taken at magnifications of 40× and 400×. Values are expressed as the mean ± SEM * P< 0.05, ** P< 0.01 and *** P< 0.001. NS is non-significant.

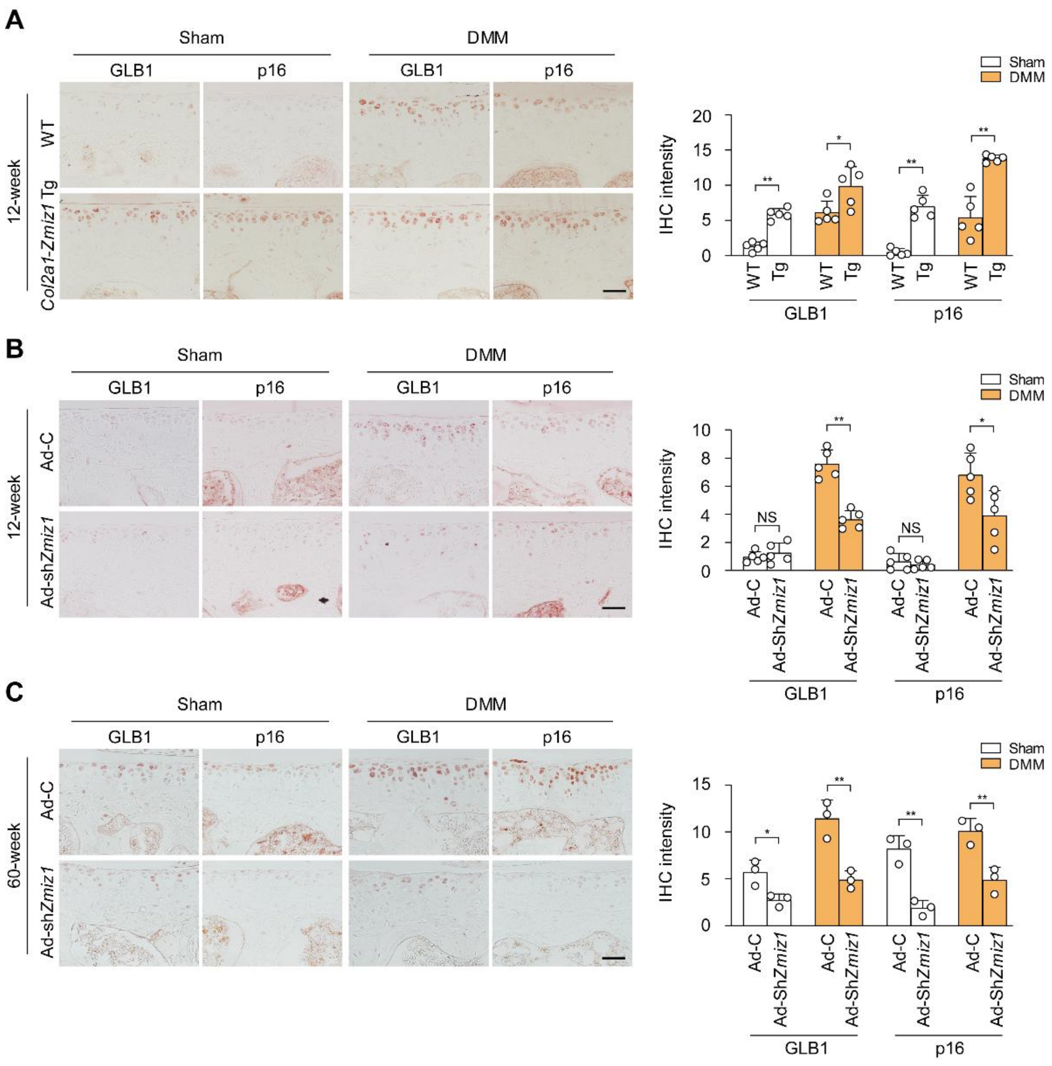

Fig. S12 ZMIZ1 regulates SASP factor expression.

(A) Detection and quantification of GLB1 and p16 in young (12-week-old) WT and *Col2a1-Zmiz1* Tg mice with DMM-induced OA (n = 5; Mann-Whitney U test). **(B)** Detection and quantification of GLB1 and p16 proteins in tissues of young mice with Ad-sh*Zmiz1*-induced *Zmiz1* knockdown (n = 5; Mann-Whitney U test). **(C)** Identification of aging markers in DMM-induced 60-week-old mice injected with Ad-sh*Zmiz1* (n = 3; Two tailed t-test). Histological image scale bar: 50 µm. IHC images were taken at a magnification of 400×. Values are expressed as the mean ± SEM * P< 0.05 and ** P< 0.01. NS is non-significant.

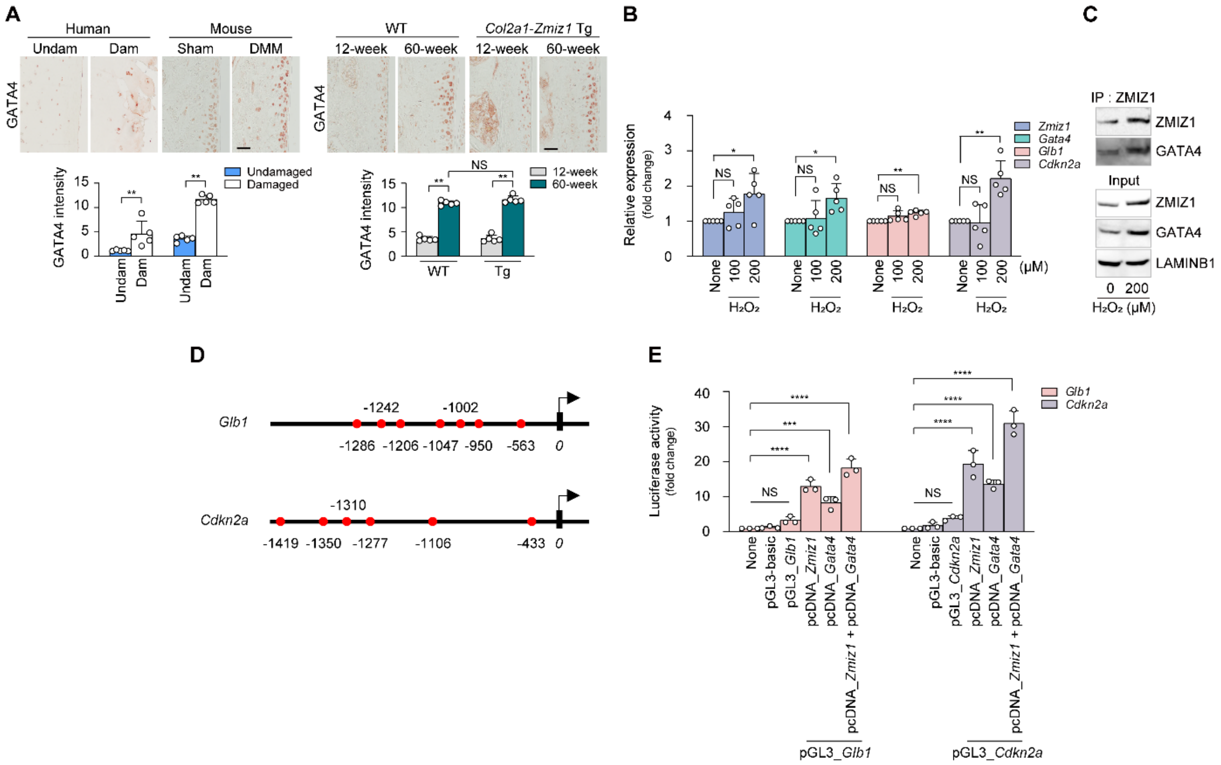

Fig. S13 GATA4 is involved in OA pathogenesis.

(A) Confirmation and quantification of GATA4 expression in damaged human and mouse cartilage (left) and in aged WT and Col2a1-*Zmiz1* Tg mice (right) (*n* = 5; Mann-Whitney U test). **(B)** Gene expression levels of *Zmiz1*, *Gata4*, and cartilage aging factors (*Glb1* and *Cdkn2a*) in chondrocytes subjected to H_2O_2-induced senescence (*n* = 5; one-way ANOVA with Dunnett's multiple comparison test). **(C)** Validation of ZMIZ1-GATA4 binding under aging conditions via co-IP (*n* = 4). **(D)** GATA4 binding regions of each gene promoter. **(E)** Luciferase activity measurement for promoter gene assay (*n* = 3; one-way ANOVA with Dunnett's multiple comparison test). IHC images were taken at a magnification of 400×. Scale bar: 50 μm. Values are expressed as the mean ± SEM * *P*< 0.05, ** *P*< 0.01, *** *P*< 0.001 and **** *P*< 0.0001. NS is non-significant.

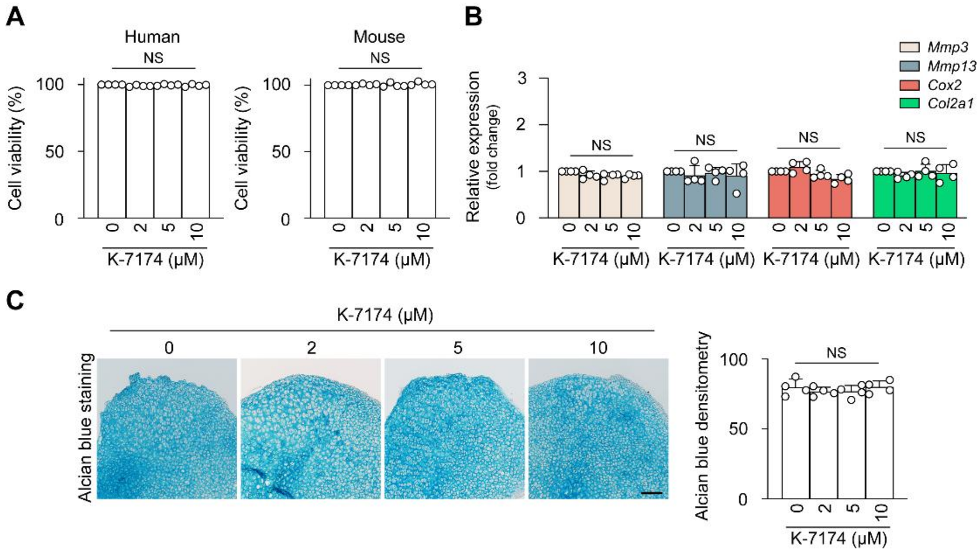

Fig. S14 Cytotoxicity of K-7174 in chondrocytes and cartilage.

To assess the potential cytotoxicity of K-7174, human and mouse chondrocytes and mouse cartilage explants were treated with K-7174 for 24 hours. **(A)** Measurement of cell cytotoxicity after K-7174 treatment in human (left) and mouse (right) chondrocytes. **(B)** Expression levels of representative cartilage differentiation factors (*Mmp3*, *Mmp13*, and *Cox2*) and an anabolic factor (*Col2a1*) in K-7174-treated chondrocytes (n = 4; one-way ANOVA with Dunnett's multiple comparison test). **(C)** Alcian blue staining and quantification of staining in cartilage explants treated with K-7174 (n = 4; scale bar: 100 μm; one-way ANOVA with Dunnett's multiple comparison test). Alcian blue staining images were taken at a magnification of 200×. Values are expressed as the mean ± SEM. NS is non-significant.

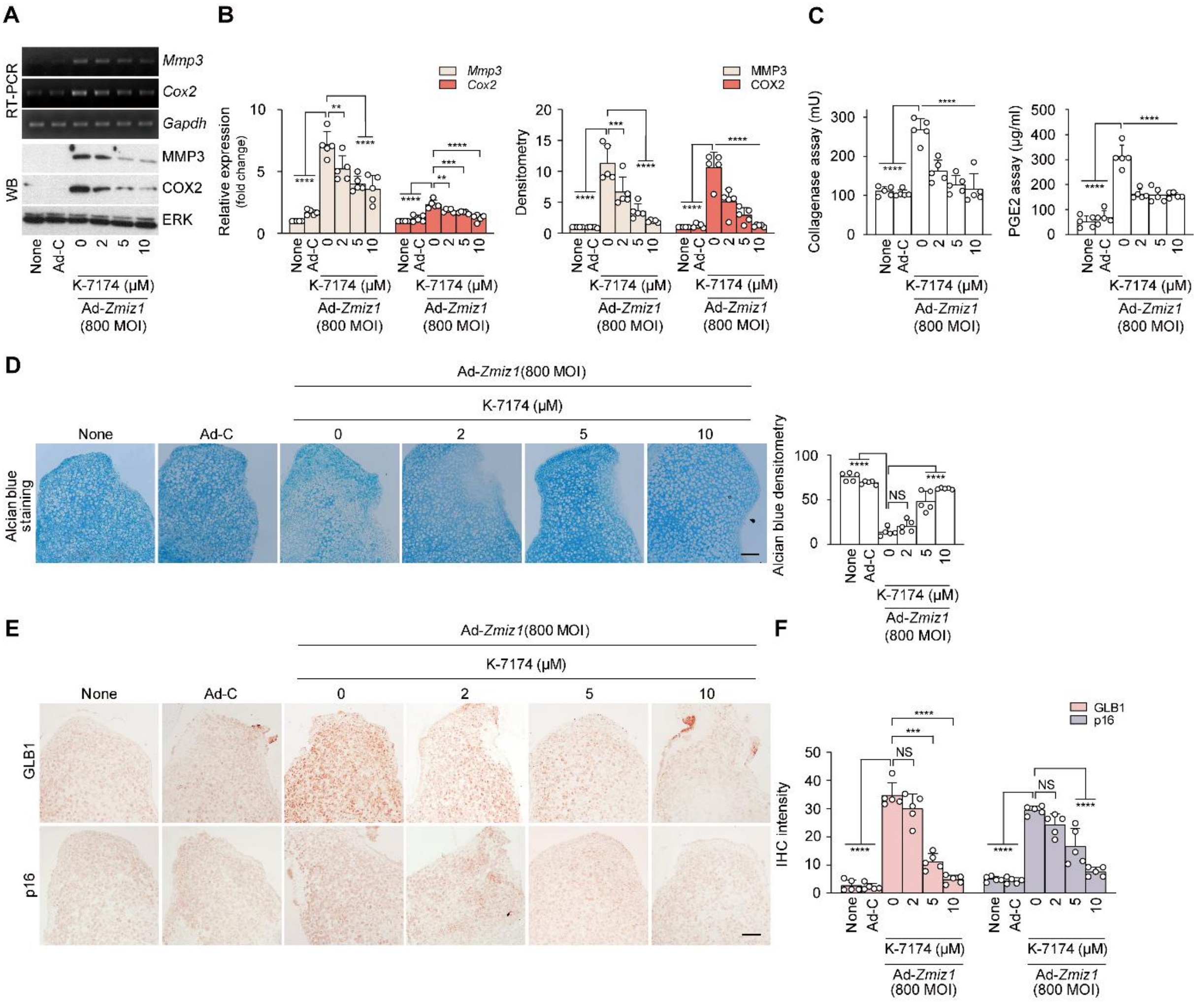

Fig. S15 K-7174 inhibits ZMIZ1-induced catabolic and senescence factor expression.

(A) The expression levels of catabolic factors in Ad-*Zmiz1* (800 MOI)-infected mouse articular chondrocytes treated with K-7174 for 24 hours, as determined by RT-PCR (upper) and Western blotting (lower) **(B)** Quantification of mRNA (left) and protein (right) levels in the chondrocytes described in (A), as assessed by qRT-PCR and densitometry (*n* = 5; one-way ANOVA with Dunnett's multiple comparison test). **(C)** Collagenase activity (left) and PGE_2 production (right) were determined following K-7174 treatment of Ad-*Zmiz1*-infected chondrocytes (*n* = 5; one-way ANOVA with Dunnett's multiple comparison test). **(D)** Alcian blue and immunohistochemical staining of Ad-*Zmiz1*(800 MOI)-infected articular cartilage explants treated with the indicated concentrations of K-7174 for 24 hours (left; *n* = 5; scale bar: 50 μm) and quantification of stained areas in cartilage explants (right; *n* = 5; one-way ANOVA with Dunnett's multiple comparison test). **(E)** Expression levels of representative SASPs (GLB1 and p16) were detected in Ad-*Zmiz1* (800 MOI)-infected cartilage explants treated with the indicated concentrations of K-7174 for 24 hours (*n* = 5; scale bar: 100 μm). **(F)** Quantification of expression in tissues (*n* = 5; one-way ANOVA with Dunnett's multiple comparison test). Explant cartilage images were taken at a magnification of 200×. Values are expressed as the mean ± SEM. ** $P < 0.01$, *** $P < 0.001$ and **** $P < 0.0001$. NS is non-significant

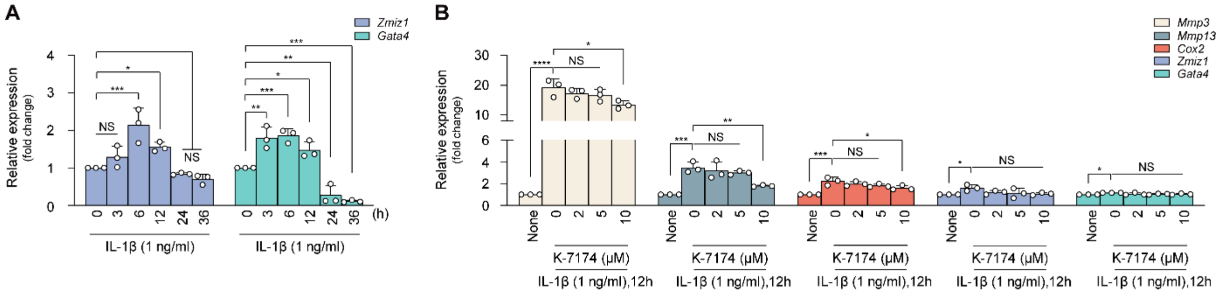

Fig. S16 K-7174 inhibits catabolic factor expression without altering the expression of *Zmiz1* and *Gata4*.

(A) Quantification of *Zmiz1* and *Gata4* in chondrocytes treated with IL-1β (1 ng/ml) (n = 3; one-way ANOVA with Dunnett's multiple comparison test). **(B)** Changes in gene expression levels of catabolic factors (*Mmp3*, *Mmp13*, and *Cox2*), *Zmiz1*, and *Gata4* in chondrocytes co-treated with K-7174 and IL-1β (1 ng/ml) for 12 hours (n = 4; one-way ANOVA with Dunnett's multiple comparison test). Values are expressed as the mean ± SEM. * $P< 0.05$, ** $P< 0.01$, *** $P< 0.001$ and **** $P< 0.0001$. NS is non-significant.

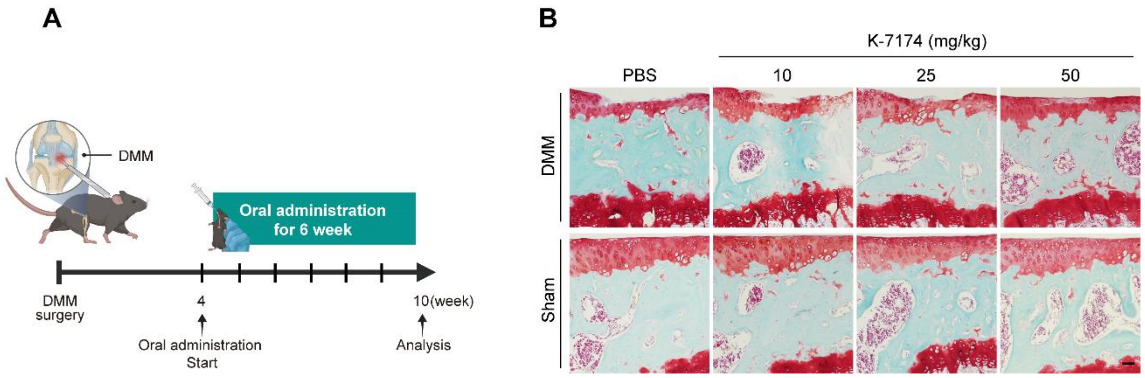

Fig. S17 Oral administration of K-7174 to DMM-induced OA mice.

(A) Schedule for oral administration of K-7174 at 10, 25, or 50 mg/kg or PBS (control). At 4 weeks after DMM surgery, PBS or the indicated concentration of K-7174 was orally administered to mice thrice per week for 6 weeks. **(B)** Subchondral bone plate image of orally administered cartilage tissue. Safranin O staining images were taken at magnifications of 200×; scale bar: 100 μm.

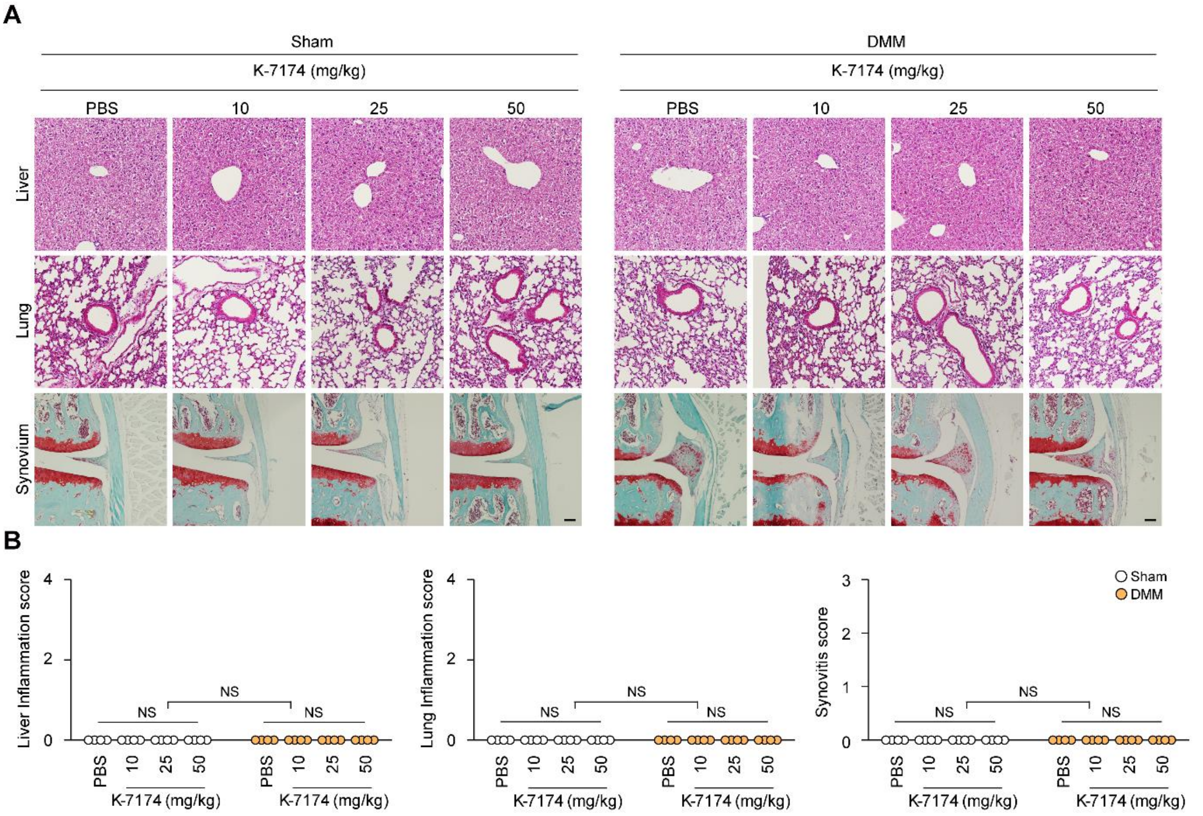

Fig. S18 Orally administered K-7174 does not trigger apparent organ cytotoxicity in DMM-induced OA mice.

(A) Histological staining of the liver, lungs, and knee joint synovium of mice treated with K-7174 for 6 weeks after DMM surgery (*n* = 4, scale bar: 100 μm). **(B)** Liver, lung, and synovial damage score measurement for the mice described in (A) (*n* = 4; one-way ANOVA with Dunnett's multiple comparison test). All histological images were taken at a magnification of 200×. Values are expressed as the mean ± SEM. NS is non-significant.

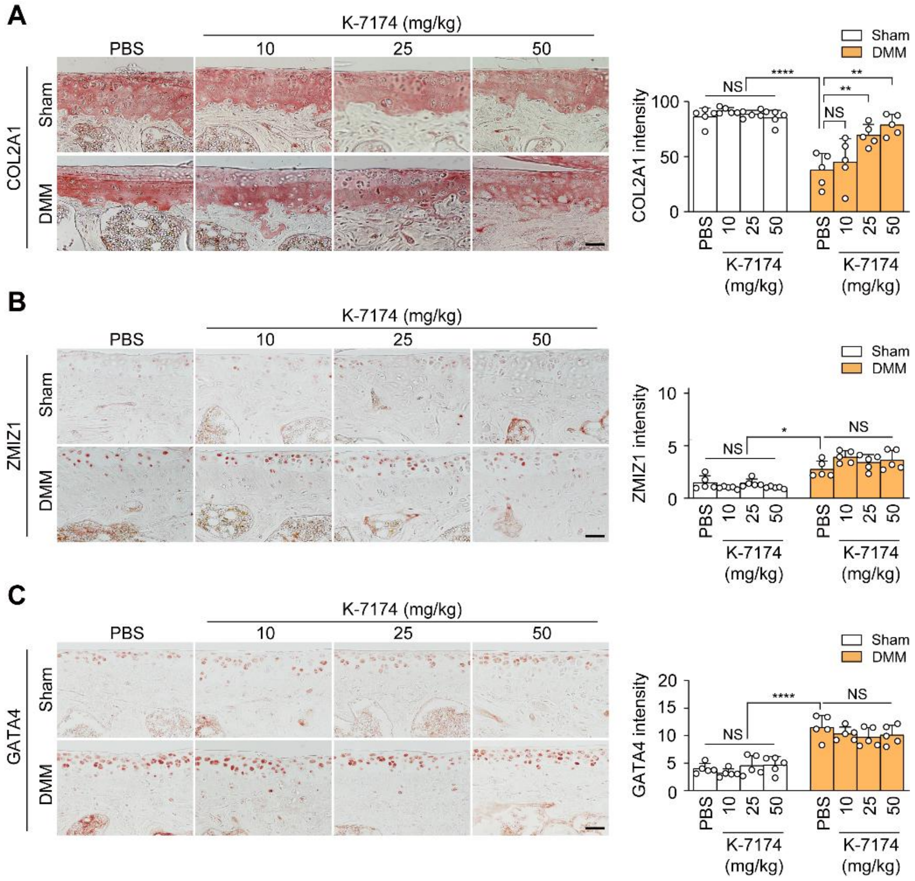

Fig. S19 K-7174 treatment recovers decreased COL2A1 expression but does not alter ZMIZ1 or GATA4 expression in DMM-induced OA mice.

(A-C) Confirmation and quantification of **(A)** COL2A1, **(B)** ZMIZ1, and **(C)** GATA4 expression in cartilage tissues of K-7174-treated mice, as assessed by IHC (*n* = 5; one-way ANOVA with Dunnett's multiple comparison test). All histological images were taken at 400×. Scale bar: 50 µm. Values are expressed as the mean ± SEM. ** *P*< 0.01 and NS is non-significant.

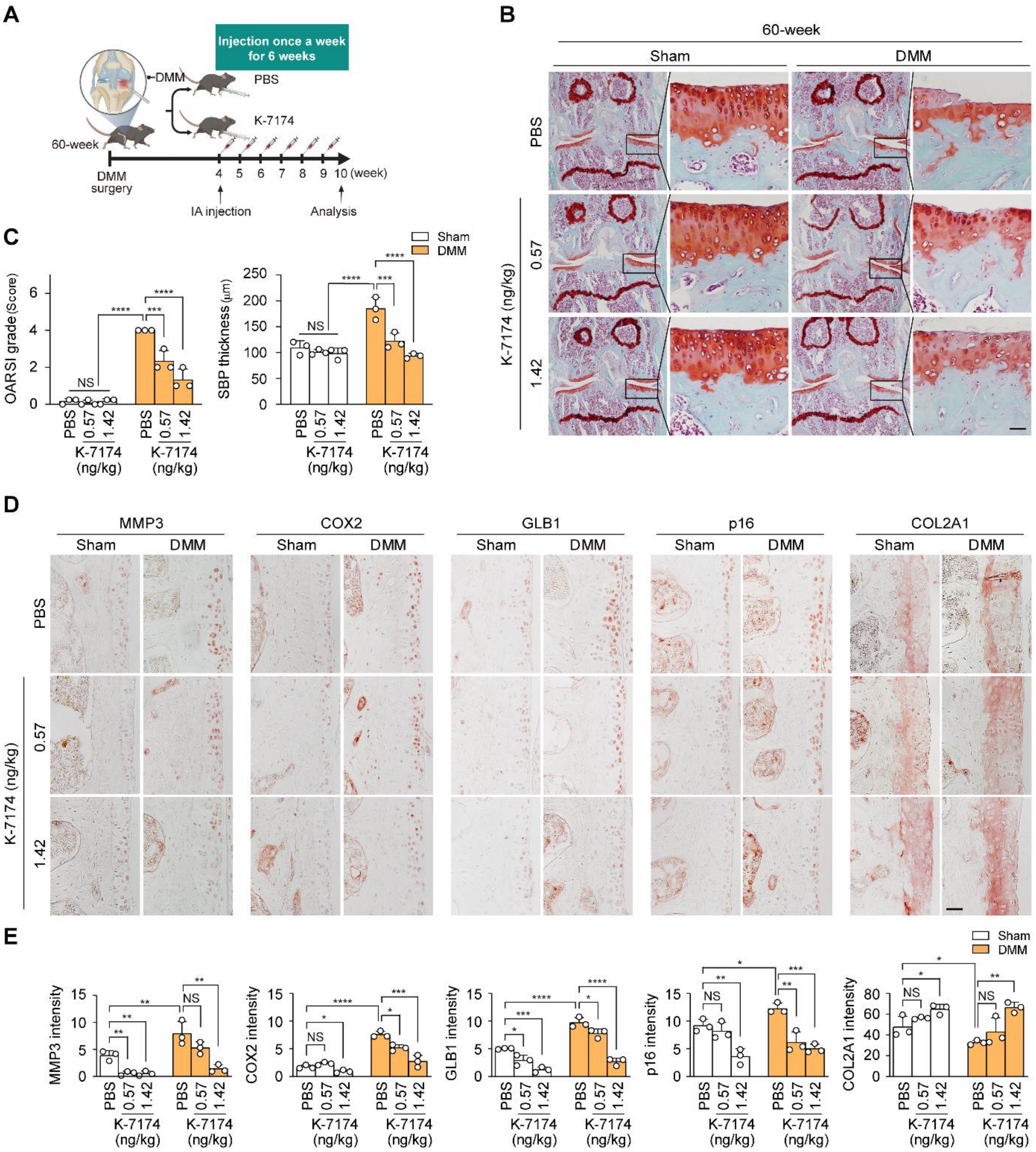

Fig. S20 Intra-articular injection of K-7174 blocks cartilage destruction in DMM-induced 60-week-old mice.

(A) Schematic diagram for IA injection of K-7174 into 60-week-old mice that had undergone DMM surgery. Mice were injected once a week for 6 weeks, starting the 4th week after surgery. **(B)** Cartilage tissues were stained with safranin O (n = 3). **(C)** Measurement of cartilage damage using OARSI grade and SBP thickness (n = 3; one-way ANOVA with Dunnett's multiple comparison test). **(D** and **E)** IHC and densitometry results for MMP3, COX2, GLB1, p16 and COL2A1 in 60-week-old WT mice (n = 3; Mann-Whitney U test). Safranin O-staining images were taken at 40× and 400×. IHC images were taken at 400×. All image scale bars: 50 μm. Values are expressed as the mean ± SEM. * P< 0.05, ** P< 0.01, *** P< 0.001 and **** P< 0.0001. NS is non-significant.

Supplemental Table

Table S1. The expression level of transcription regulator top 50.

Transcription regulator merges top 50.			
Gene	Fold change	12-week-old	60-week-old
PLAG1	6.51	62.48	406.59
HDGF	5.12	218.38	1118.46
TET3	4.97	79.62	395.78
TBX15	4.69	434.56	2038.7
CBFA2T2	4.47	92.27	412.46
SOX11	4.31	172.21	742.16
ETV5	4.26	133.72	570.14
TOX4	4.23	45.92	194.12
BCL3	4.2	281.16	1181.66
NFIB	4.11	98.84	406.38
MIER1	3.95	238.61	942.5
RBM14	3.88	169.45	657.15
HMGA1	3.78	394.41	1490.29
NAB2	3.73	430.4	1606.38
PHB2	3.67	340.69	1251.45
CALCOCO1	3.65	257.91	941.47
NOTCH1	3.65	203.67	743.16
KLF3	3.61	278.57	1004.69
RAD54L2	3.52	58.48	206
PEG3	3.5	1977.11	6928.41
NFKBIB	3.47	388.34	1347.96
NONO	3.42	769.12	2628.75
ANKRD49	3.41	161.51	550.16
RUNX1T1	3.4	113.78	386.57
ASB4	3.32	81.04	268.86
FOXP2	3.23	145.69	470.82
ZFP57	3.2	374.01	1196.88
FOXA3	3.19	227.82	726.61
HCFC1	3.19	205.09	655.04
NPAS4	3.14	155.93	490.37

ZMIZ1	3.14	1258.4	3946.56
SREBF2	3.12	426.64	1332.49
TARDBP	3.01	122.24	367.73
SIX1	3	147.08	441.05
RELB	2.9	118.23	342.7
GTF2IRD1	2.89	204.36	590.13
KLF12	2.87	232.81	668.2
SMARCA1	2.87	119.63	343.6
UBE2V1	2.86	353.52	1010.67
MTA2	2.8	173.76	485.99
NPAS3	2.79	110.48	308.7
TRRAP	2.79	255.54	712.63
ARID2	2.75	281.68	774.32
CREB3L1	2.75	396.44	1089.33
GTF3C3	2.75	79.61	218.56
SRCAP	2.75	371.28	1020.63
FOXP4	2.74	231.74	635.22
CSRNP1	2.72	264.09	718.2
PTPN14	2.68	316.95	850.6
SKI	2.68	540.48	1451.05

Table S2. Characteristics of OA patients from whom specimens were obtained.

No.	Age/gender	ICRS[a] grade	Joint	Weight (kg)	Height (m)	BMI[b] (kg/m^2)
1	65/F	4	Knee	158	53	21.23
2	80/F	4	Knee	143.3	55.1	26.83
3	72/F	4	Knee	165	65	23.88
4	63/F	4	Knee	152	52	22.51
5	69/F	4	Knee	151	60	26.31
6	73/F	4	Knee	153.8	70.75	29.89
7	63/F	4	Knee	156	72.2	29.67
8	73/F	4	Knee	154	83	35
9	75/F	4	Knee	154	55	23.19
10	63/F	4	Knee	163	74.3	27.96

[a]ICRS, International Cartilage Repair Society; [b]BMI, Body Mass Index

Table S3. Primer sequences and conditions of RT-PCR and qRT-PCR.

Gene	Origin	Strand	Sequence	Size (bp)	AT (°C)
Zmiz1 *(RT and qRT)*	Mouse	S	5'-CCCCGCCAACTTCCACAAT -3'	280	60
		As	5'-GTTCTGGCGGCTCTTCTGA -3'		
Mmp3 *(RT)*	Mouse	S	5'-CTGTGTGTGGTTGTGTGCTCATCCTAC-3'	350	58
		As	5'-GGCAAATCCGGTGTATAATTCACAATC-3'		
Mmp3 *(qRT)*	Mouse	S	5'-TCCTGATGTTGGTGGCTTCAG -3'	102	60
		As	5'-TGTCTTGGCAAATCCGGTGTA -3'		
Cox2 *(RT)*	Mouse	S	5'-GGTCTGGTGCCTGGTCTGATGAT-3'	724	63
		As	5'-GTCCTTTCAAGGAGAATGGTGC-3'		
Cox2 *(qRT)*	Mouse	S	5'-TTCAACACACTCTATCACTGGC -3'	271	60
		As	5'-AGAAGCGTTTGCGGTACTCAT -3'		
Gapdh *(RT and qRT)*	Mouse	S	5'-TCACTGCCACCCAGAAGAC-3'	450	58
		As	5'-TGTAGGCCATGAGGTCCAC-3'		
Zmiz1	*Zmiz*Tg genotyping	S	5'-GACCTGACTCTGGGATGTGTT -3'	714	60
		As	5'-ACTACACCCTGGTCATCATCC -3'		
Cdkn2a *(qRT)*	Mouse	S	5'-CGTACCCCGATTCAGGTGAT-3'	245	60
		As	5'-ACGATGTCTTGATGTCCCCG-3'		
Glb1 *(qRT)*	Mouse	S	5'-TTTCTGGGGACCGTGATGTG-3'	247	60
		As	5'-ATCGGTCCTCCGTTCTGGTA-3'		
Col2a1 *(qRT)*	Mouse	S	5'-CACACTGGTAAGTGGGGCAAGA-3'	173	60
		As	5'-GGATTGTGTTGTTTCAGGGTTCG-3'		
Aggrecan *(qRT)*	Mouse	S	5'-GAAGACGACATCACCATCCAG-3'	581	60
		As	5'-CTGTCTTTGTCACCCACACAT-3'		
Sox9 *(qRT)*	Mouse	S	5'-GAGCCGGATCTGAAGAGGGA-3'	132	58
		As	5'-GCTTGACGTGTGGCTTGTTC-3'		
Mmp3 ChIP *(qRT)*	Mouse	S	5'-AAGCGGGGAATGATAGCCTG-3'	545	63
		As	5'-TTGGCCAGAGACCGAACATC-3'		
Cox2 ChIP *(qRT)*	Mouse	S	5'-TTTTGAGCAGGGGTCTTGCT-3'	566	63
		As	5'-TGCTGAGCTCCACTTCATCG-3'		
Glb1 ChIP *(qRT)*	Mouse	S	5'-TTCAAATTTTGGGCTGGGCG-3'	540	63
		As	5'-AGCTGGCCTGGAACTCTAGA-3'		
Cdkn2a ChIP *(qRT)*	Mouse	S	5'-TGGCTTCTGTTCAGTTGCCA-3'	599	63
		As	5'-GCTCATCCCAGCACTTAGGG-3'		

AT, annealing temperature; S, sense primer; As, antisense primer
